# Partisanship and Covid-19 vaccination in the UK

**DOI:** 10.1038/s41598-022-23035-w

**Published:** 2022-11-18

**Authors:** Margaryta Klymak, Tim Vlandas

**Affiliations:** 1 Somerville College, University of Oxford, Woodstock Rd, Oxford, OX2 6HD UK; 2 Department of Social Policy and Intervention, University of Oxford, 32 Wellington Square, Oxford, OX1 2ER UK

**Keywords:** Public health, Health care, Vaccines

## Abstract

This article examines the association between partisanship and vaccination in the UK. The lower vaccination rates among Republicans in the US have been linked to ideology and President Trump’s anti-vaccination rhetoric. By contrast, both ruling and opposition parties in the UK promoted the national vaccination program. Using two datasets at constituency and individual levels, we analyse whether there are partisan differences in uptake when vaccination garners cross-party support. Our findings contrast in important ways from the US case. First, the correlation between partisanship and vaccination is the opposite to that of the US: both Conservative constituencies and individuals are associated with higher vaccination rates than Labour across almost all age groups. Thus, right-leaning individuals do not necessarily vaccinate less, at least when their political party is in power and supportive of vaccination. Second, partisanship alone accounts for a large share of variation in vaccination rates, but this association appears largely driven by socio-economic and demographic differences: older and economically better off individuals and constituencies tend to be more vaccinated. Once these controls are included, the correlation between Conservative partisanship and vaccination shrinks substantially. Hence, the ideological source of the partisan gap in vaccination rates appears smaller than in the US.

## Introduction

Covid-19 has caused over 6.4 million deaths worldwide^1^ and resulted in profound economic, political and social consequences. The main policy to prevent further infections and deaths is vaccination. Yet, many individuals remain reluctant to vaccinate, even in places with widely available and free vaccines. A prominent example is the US, where partisanship has been shown to shape vaccination rates with Republican individuals and States tending to vaccinate less^2–6^. This partisan difference echoes a wider literature arguing that societies are politically polarized, with voters of various political parties often thinking and behaving differently^7,8^. As a result, this difference in vaccination between Republicans and Democrats is often interpreted as evidence that right-wing ideology leads to lower willingness to vaccinate. However, it is in practice challenging to disentangle the effects of Republican voters’ ideology from the position of their leader in government, the Covid-19 sceptic and anti-vaccination President Trump^9,10^, since both overlapped in the US case.

Thus, in this article we focus on another prominent democracy, the UK, that shares two important similarities with the US case, but differs in one crucial way, which makes it possible to sidestep this aforementioned overlap in ideology and leader’s position. The first similarity concerns the severity of the Covid-19 pandemic. Second, the extent of partisan polarization has also been widely documented in the UK^11,12,13^. The crucial difference between the US and UK concerns the Covid-19 position of political parties and the elected government. It is a priori not clear whether the association between partisanship and vaccination should be driven by similar dynamics in the UK, where both mainstream parties converge on this aspect and the country is led by a pro-vaccination right-wing government. In this article, we ask whether there are partisan differences in vaccination uptake between voters when vaccination is not politicized along partisan lines by different political parties?

We explore this question empirically by analysing whether and how partisan dynamics help us make sense of variation in vaccination rates in England using two separate datasets. First, we combine the latest general election results with vaccination data reported by the National Health Service (NHS). In contrast to the US case, we show that the Conservative vote share across English constituencies is positively correlated with higher rates of vaccination. However, this correlation appears largely, although not fully, driven by economic and socio-demographic differences between constituencies: older and economically better off constituencies tend to be more vaccinated, and once these controls are included, the association between Conservative vote share and vaccination shrinks substantially, although it remains statistically significant.

Second, we analyse an individual level dataset collected by YouGov in October and November 2021. This analysis confirms that Conservative voters are not less likely to vaccinate than Labour voters, again in contrast to findings in the US. If anything, the correlation between individual past Conservative vote and vaccination is positive and statistically significant, although it is not substantial. The individual level partisan gap in vaccination likelihood also shrinks when more characteristics are included, consistent with the notion that partisan differences might be smaller in the UK than in the US. We speculate this may be due to the greater pro-vaccination position of the Conservative UK government.

While our results remain correlational, we aim to describe how and whether the direction, statistical significance, and magnitude of the association between voting and vaccination varies when controlling for different factors. Partisan differences across English constituencies and individuals are mostly capturing economic and socio-demographic factors. We cannot infer from this correlational analysis whether the remaining statistically significant differences in vaccination have ideological sources, nor whether these sources have a causal effect. Instead, the importance of our findings is that they demonstrate that right-leaning individuals do not necessarily and always vaccinate less, so the findings from the US case^14–19^ cannot be extrapolated to other contexts. Thus, partisan patterns in vaccination appear to depend on the country-specific position of different political parties and the government.

## Context and background

### The case of the UK

In this article we focus on the case of the UK because it has two main similarities with the US case, but differs in one important way, which makes it possible to sidestep the overlap in ideology and leader’s position in the US case. The first similarity concerns the severity of the Covid-19 pandemic. At the time of writing, the UK had over 19 million confirmed cases and nearly 159,000 deaths, making it one of the most affected countries by the Covid-19 pandemic. Like many other governments across the world^20^, the UK had initially attempted to contain Covid-19 with a national lockdown^21^, but then eventually started to relax these restrictions. While these measures were found to be effective at reducing transmission^22–24^, the timing and precise forms of these interventions were heavily politicized and widely debated, especially between different political parties in parliament.

The second similarity relates to the extent of partisan polarization in the US, which has also been widely documented in the UK^11,25^. Debates have focused on whether distinct voter groups have systematically different attitudes, behaviours, and policy priorities, and if so, what can explain these differences^26^. Partisan ideology has notably been argued to shape how people respond to scientific facts^27^. Yet, the emerging literature on the characteristics associated with vaccination in the UK tend to focus on psychological, social, and economic factors^28–31^ without considering the possible relationship between partisanship and vaccination found in the US case^32^.

A key difference between the US and UK relates to political parties and the elected government. Right-wing voters were less likely to vaccinate in the US, and at least some parts of the Republican party—including President Trump—were Covid-19 sceptic and anti-vaccination^33,34^. By contrast, the Conservative government led and actively promoted the vaccination campaign^35,36^ and the consensus on vaccination in the UK was much more bipartisan given Labour’s support^37,38^. Hence, it is a priori not clear whether the association between partisanship and vaccination should be driven by similar dynamics in the UK. Our research question is therefore whether any partisan differences in vaccination rates can be observed when both mainstream parties converge on this aspect and the country is led by a pro-vaccination, right-wing government.

### Partisanship, Covid-19, and vaccination

An emerging literature considers the association between partisan ideology and Covid-19 attitudes and behaviours. In terms of ideology, most studies suggest that right-leaning individuals are less likely to vaccinate. In the US, Republicans are less likely to comply with government Covid-19 interventions, such as social distancing and mask wearing, and are more Covid-19 and vaccine sceptic ^39–43^. Several reasons for this association have been explored. A first set of reasons include differential conspiracy theory beliefs and anti-intellectualism among Republican voters^44^. Libertarianism was also found to predict lower support for vaccine mandates in the United States^45^. Another set of reasons relates to education and knowledge of Covid-19 as key factors shaping vaccine hesitancy^46^. In the US, right-wing respondents are less anxious about the Covid-19 pandemic compared to left-wing respondents^47^.

Studies in other countries confirm some of the partisan correlations with vaccination and Covid-19 attitudes found in the US case. Right-wing political position or ideology is positively associated with vaccine hesitancy in Austria^48^, Norway^49^, and New Zealand^50^. Populist orientation is correlated with vaccine refusal in Italy, but is moderated by perceived vulnerability to Covid-19 infection^51^. Perceived vulnerability of falling ill to Covid-19 strongly moderates the positive effect of populist attitudes on vaccine refusal^52^. A multi-country experimental study in seven European countries finds that left-leaning individuals are more likely to favour strict Covid-19 health measures, as they are “traditionally more in favour of a proactive role of the state in providing citizens’ wellbeing in the name of solidarity and traditionally supported by social categories having an easier access to economic relief during the Covid-19 crisis”^53^.

Thus, if findings and logics documented in other countries travel to the UK case, given differences in ideology between Conservative and left-wing voters, one would expect that Conservative voters are more Covid-19 sceptic, less likely to vaccinate, less likely to favour strong state interventions, more likely to prioritize the economy if there is a trade-off with public health, more likely to believe in conspiracy theories, and more likely to oppose Covid-19 measures.

Yet, two other perspectives in the literature yield more complex predictions about the relationship between partisanship and vaccination. A first perspective focuses on material differences. In the UK, Conservative voters tend to be older, more economically secure and higher socio-economic occupation, while Labour voters tend to be younger and more educated^54^. The characteristics of Conservative voters, specifically being older and having more wealth, make them more likely to have accessed the vaccine at earlier stages of its rollout. On the other hand, the greater presence of Labour voters among large urban centres^55^ could play both ways: large cities might have better vaccination infrastructure, but could also face greater population pressures. Thus, in this first perspective, after we account for these observed socio-economic and demographic characteristics, there might not be any differences in vaccination rates between Conservative and Labour voters.

Second, it is also possible that partisan effects depend crucially on whether their preferred party is in power enacting Covid-19 policies and what this political party (and their elected representatives) say to their electorate. Voters and supporters of opposition parties report higher vaccine hesitancy in several countries. For instance, in France, vaccine hesitancy is lower among those who are close to ruling parties than among people who support far-left or far-right parties^56^. In Austria, this is largely explained by lower trust in authorities^57^. In the US, cues from elite Republicans endorsing vaccination are associated with an increase in vaccination intent among Republicans compared to receiving a cue from an elite Democrat. By contrast, Republicans who received a Democrat elite cue on vaccine endorsement were less likely to become vaccinated^58^.

 In the UK, it could therefore be that Conservatives vaccinate more because they ‘follow their leader’, and hence are more compliant with authority, or because they have greater trust in the government. Crucially, in the UK case, Prime Minister Johnson was in many instances very clearly in favour of vaccination. For instance, in his New Year’s Address, Boris Johnson declared that “there is one reason—one overriding reason—why the UK has been able to maintain the most open economy and society of any major European economy. And that is because the British people have responded heroically, voluntarily, and in almost incredible numbers to the call to get vaccinated…. And I want to speak directly to all those who have yet to get fully vaccinated. The people who think the disease can’t hurt them—look at the people going into hospital now, that could be you. Look at the intensive care units and the miserable, needless suffering of those who did not get their booster, that could be you”^59^.

## Results

We discuss the results for constituency and individual level data, respectively, in the next two sub-sections, while a more detailed methodology can be found after the discussion section.

### Vaccination rates and partisanship across constituencies in England

**Figure 1:**
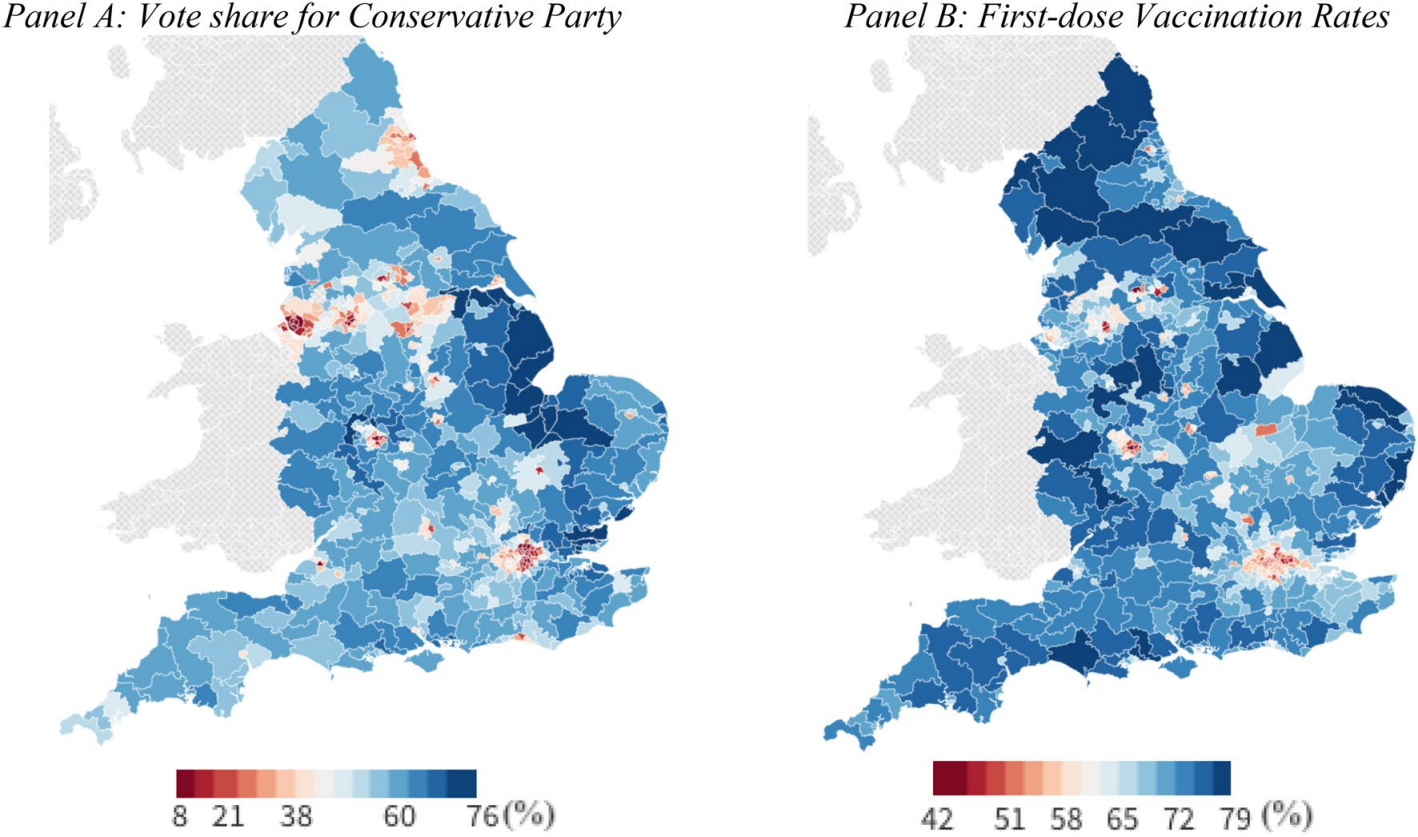
Mapping vote share and vaccination rates across English constituencies. This figure shows the vote share (left-hand side) and vaccination rate (right-hand side), both expressed as percentage points, in different English constituencies. Vaccination rates were calculated by dividing the number of first doses in constituency *i* by the total population of that constituency and then multiplying this ratio by 100. This figure was created with https://flourish.studio/.

We match constituency-level vaccination data collected by the NHS in late October 2021 to the latest general election results for all the main political parties: Conservatives, Labour, Brexit, Liberal Democrats and Greens. Figure 1 plots the geographical variation in Conservative vote shares and vaccination rates (both in percentages) across English constituencies. One can observe that many constituencies where the Conservative party received fewer votes also have lower vaccination rates. A simple ordinary least squares regression of vaccination rates on the vote share in different English constituencies suggests a strong positive *descriptive* bivariate association. The coefficient for this regression (0.295—with a 95% confidence interval of 0.266 to 0.325) implies that a one-point increase in Conservative vote share is associated with 0.295 percentage point higher vaccination rates (second dose). Differences in Conservative vote share account for 42% of the variation in vaccination rates in second doses in England.

**Figure 2:**
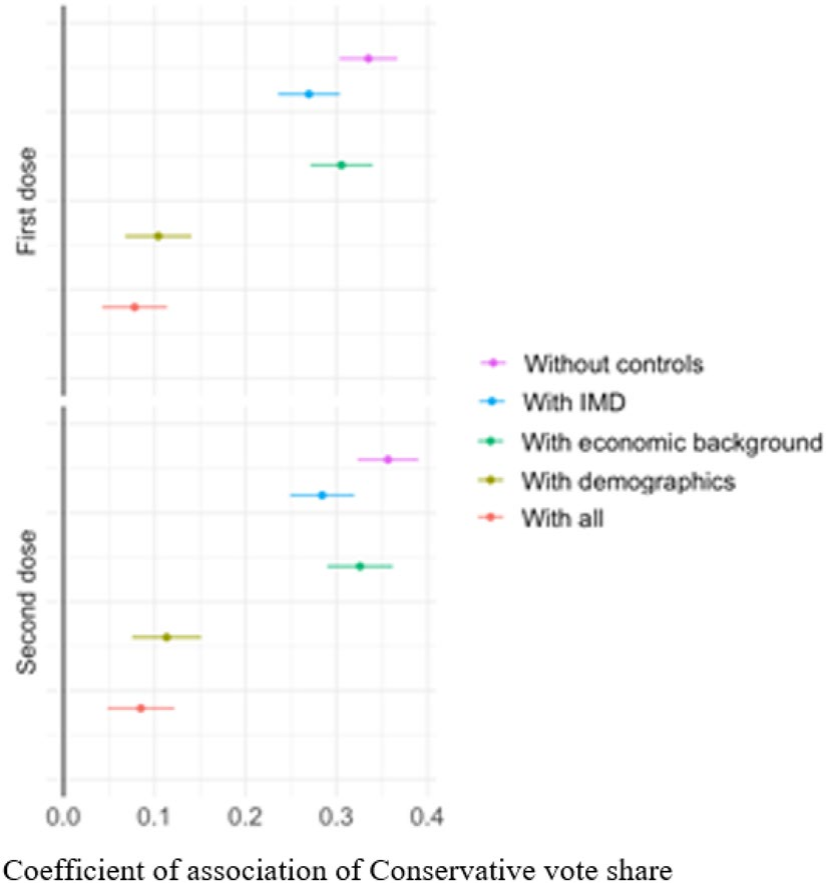
Vaccination and conservative vote share. This figure shows the correlation between the Conservative share of votes and vaccination rates across constituencies. Each circle captures the coefficient of association of Conservative vote share, with the bar on either side showing 95% confidence intervals. The association is statistically significant when the confidence interval does not overlap with the 0-line represented by the vertical axis. The x-axis shows the size of the effect of a one percentage point increase in Conservative vote share on a percentage point increase in vaccination rate. Different colours capture whether the results are based on regressions, without or with a different set of controls. The regression without any controls has a sample size of 532 observations. Once all controls are included, this shrinks to 463 observations. For full results and robustness checks, please see Section S1 in supplementary information.

While this strong geographical partisan divide in vaccination rates represents a challenge to achieving full vaccination of the population, many confounding factors may be captured by the coefficient for Conservative vote share, and these confounding factors therefore need to be controlled for. To address this issue, we perform a series of regressions described in the Methods section. Figure 2 plots the coefficients capturing the association between the share of Conservative vote and vaccination rates for first and second doses, respectively. Our main result is that the share in vaccination rate is positively correlated with the share of votes that the Conservative party received in the last national election.

As we include more controls to capture potential confounding factors, the coefficient of association between Conservative vote and vaccination rates shrinks, consistent with the notion that bivariate partisan differences conceal other socio-economic and socio-demographic factors, but it remains statistically significant. When focusing on the first-dose vaccination rate, the coefficient for Conservative votes is 0.078 and statistically significant (with a 95% confidence interval of 0.042 to 0.114), while for second-dose vaccination rate, the coefficient is 0.085 and statistically significant (with 95% a confidence interval of 0.048 to 0.122).

In our regressions on second-dose vaccination rates, we find that socio-economic factors matter: the coefficient for median house prices is −0.008 (with a 95% confidence interval of −0.013 to −0.004) while the coefficient for median wages is -0.166 (with a 95% confidence interval of −0.274 to −0.059). Some of the socio-demographic factors also have an association with the vaccination rate: the coefficient is 0.290 (with 95% a confidence interval of 0.236 to 0.344) for the variable measuring the ethnic composition of a constituency, whereas it is 0.091 for population density (but not statistically significant since the 95% confidence interval is −2.159 to 2.341). The coefficient for an index of multiple deprivation (IMD) is 0.0211 (with a 95% confidence interval of 0.018 to 0.024).

**Figure 3:**
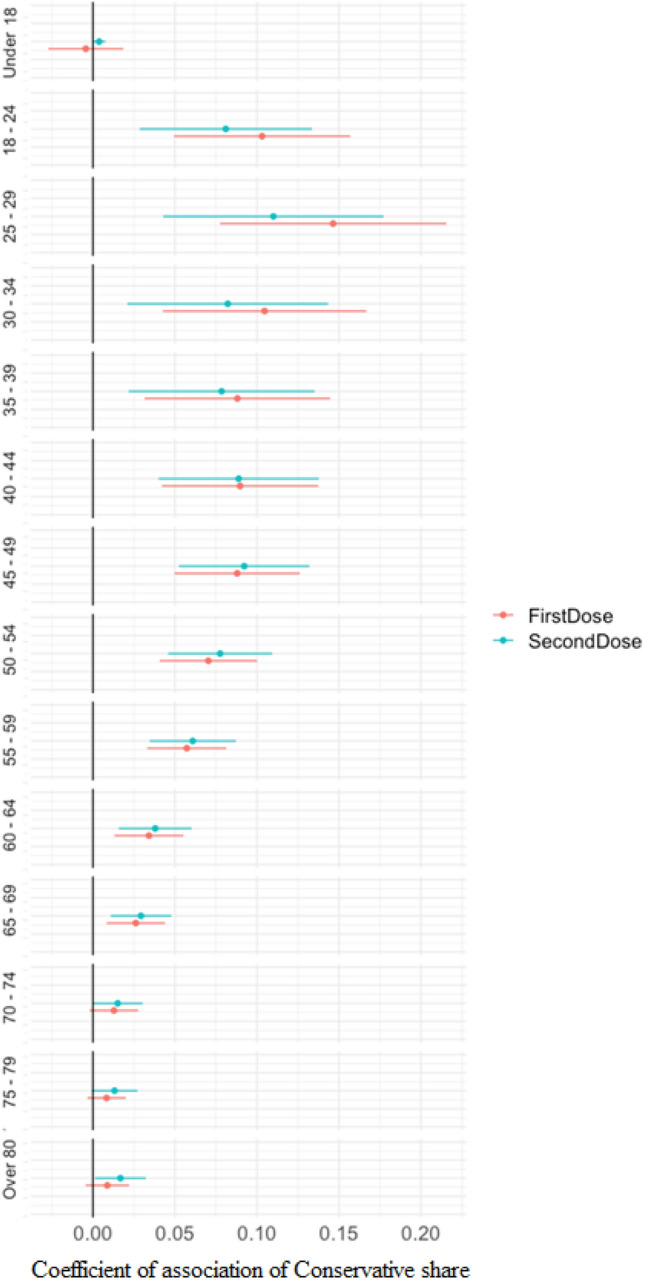
Vaccination for different age groups and conservative vote share. See notes of Fig. 2. Different colours capture results for first versus second dose vaccination rates, respectively.

When restricting the sample for each age group, the coefficient for conservative vote share remains positive and statistically significant for almost all age groups (Fig. 3). Leaving aside the case of those under-18 who were by and large not eligible at the time of writing, there is a clear falling association for older age groups. In other words, as the age-related risk of serious infection rises, the partisan association shrinks almost completely.

**Figure 4:**
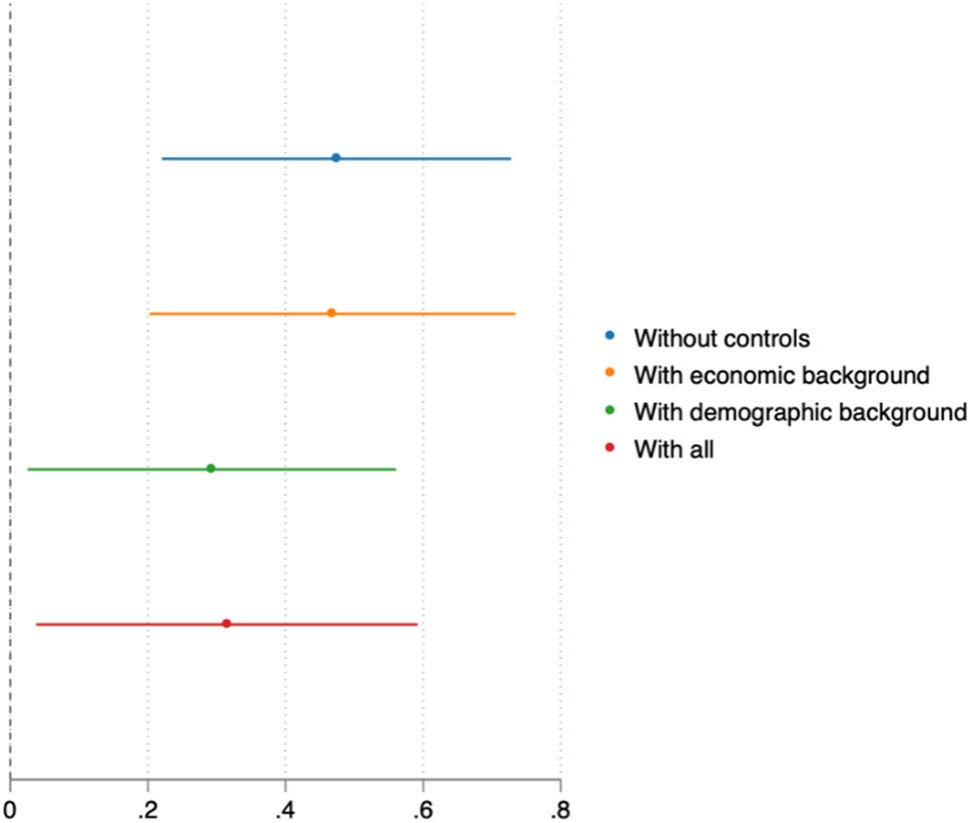
The association between being a conservative voter and being vaccinated. This figure plots the coefficients capturing the relationship between being a Conservative voter and vaccination status based on logistic regressions using five waves of a YouGov survey conducted between the 4th of October and the 9th of November, 2021. It adds controls for socio-economic background (number of children and social class), demographic characteristics (age, gender) and region fixed effects. The 95% confidence interval is calculated using robust standard errors. The sample size is 5967 observations when all controls are included.

Finally, we analyse individual-level responses to a YouGov survey conducted between 4th October and 9th November 2021. The findings shown in Fig. 4 confirm the presence of a partisan gap at the individual level: conservative voters are more likely to be vaccinated even when controlling for a wide range of relevant socio-economic and demographic factors. However, the material sources of this partisan gap become apparent when we control for all relevant socio-economic and demographic characteristics: the effect shrinks to 0.315 with a 95% confidence interval of 0.037 to 0.592.

## Discussion

This article examines whether and how partisanship correlates with variation in vaccination rates in England. Our research follows recent literature that explores differential behaviour during pandemics across voter groups^60–63,13^. Our findings contribute to emerging literature on the importance of partisanship in shaping Covid-19 perceptions and behaviours. Compared to existing studies, we find, first, that partisan differences do not strongly relate to vaccination behaviours once socio-economic and demographic factors are considered, and second, where there is a partisan effect it runs counter to that reported in other cited studies: Conservative constituencies do not appear to vaccinate less. Of particular note, our research contrasts markedly with the well-documented partisan and Covid-19 trends observed in the US where Republicans are less likely to socially distance and wear masks, but more likely to perceive Covid-19 as a minor threat and endorse anti-vaccine misinformation than their Democrat counterparts^16,43,64–67^.

Although our study therefore shows how the UK differs from the US case, it cannot definitely answer the question of why. However, we can offer a number of plausible reasons. First, we show that part of the partisan difference in the UK is driven by wealthier areas of the country also being more likely to have vaccination uptake. One can speculate that wealth removes certain material barriers to getting vaccinated, for instance by lowering the relative perceived costs of travelling to the vaccination centre and arranging child care, and providing more flexibility to miss work. Some of the partisan difference however remains even when controlling for wealth and other socio-demographic factors.

Second, the location of right-wing voters in the US and the characteristics of the US welfare state may also partly account for the differential partisan patterns to the UK. Indeed, in contrast to the NHS in the UK, the US has no national healthcare system and there is wide variation in access to healthcare. Republican States tend to be more rural (and sometimes more deprived), while densely-populated urban centres are more likely to vote for the Democratic Party^68^. Linked to this, the UK vaccination program was designed to ensure availability across the country and with particular attention paid to local access. In addition, Republican States also have fewer social programs that help people with health-related issues and have higher mortality rates, both generally and during the pandemic specifically^69^. By contrast, social policies across UK constituencies are often set at the national level, and in cases where local councils are responsible, more Conservative constituencies also tend to be wealthier and hence better resourced.

Finally, one alternative source of this partisan difference might be ideological: conservative voters may have different views towards health, the state, the economy, or indeed the importance of science than Republicans in the US, which may then lead them to vaccinate more. Another related possibility lies with the potentially strong attention that voters pay to the messages and positions of their elected representatives. If this is true, then Conservative voters may be more likely to pick up and listen to cues from Conservative Members of Parliament, Conservative ministers and Boris Johnson’s pro-vaccine messaging. To the extent that voter groups are influenced by the positions of their elected representatives, in line with existing causal evidence that voters’ opinions are shaped by the position of their political party^70^, this smaller and reversed partisan gap in the UK is consistent with the much more pro-vaccination policy stance of the Conservative government in the UK compared to the US under Trump.

Our findings have implications for increasing the ability and willingness of individuals to vaccinate. Since socio-economic determinants are important drivers of vaccination obstacles and hesitancy, governments are well advised to further incentivize individuals to vaccinate, for example by providing benefit support to allow people to take time off work to recover from the side effects of the vaccine. In addition, and in contrast to the US case, the substantially smaller partisan difference in the UK emphasizes that ideology depends strongly on the country context and that trust in a government that one elected and that supports the policy may be more important for individuals’ vaccination decisions than previously anticipated.

## Methods

In analysing partisan differences in vaccination rates, three sources of bias are possible: *one*, individual responses might suffer from a social desirability bias whereby individuals have an incentive to misreport their vaccination status to conform with societal pressures and norms; *two,* aggregate-level data is subject to an ecological fallacy so one should not infer individual-level behaviour from aggregate level patterns; *three*, omitted variable bias can lead to seemingly partisan differences that in fact conceal socio-economic and demographic factors.

To address these issues, we carry out two separate analyses of two datasets at the constituency and individual levels, respectively. First, we use constituency-level vaccination rates that are less susceptible to social desirability bias than the individual’s declared vaccination status found in surveys. Second, to address the ecological fallacy inherent in aggregated data, we analyse an individual-level dataset collected by YouGov.

### Constituency level analysis

We match constituency-level vaccination data with the latest general election results in 463 constituencies in England and then apply an ordinary least squares method using the following specification:

$${Vaccination}_{ir}={\beta }_{0}+ {\beta }_{1}{Conservative}_{ir}+ \Omega {X}_{ir}+ {\alpha }_{r}+{\varepsilon }_{i}$$

The dependent variable in this analysis is the vaccination rate (that is, the number of doses provided in constituency *i* in region *r* divided by the constituency’s population, which is publicly available from the NHS). Our regressor of interest is the vote share for the Conservative party in the latest general election in the UK, which is publicly available from the House of Commons Library.

Our analysis controls for a range of relevant background characteristics, which we introduce into our regression stepwise. First, we add the index of multiple deprivation (IMD), which is the official measure of relative deprivation in England that we obtained from the House of Commons Library. It consists of seven weighted domains of deprivation: income, employment, health deprivation and disability, education, skills training, crime, barriers to housing and services, and living environment. Second, we include two types of economic background factors: the median wages and house prices, both obtained from the Office of National Statistics. Third, we add demographic controls: we calculate the population density (in 1000s) defined as the population of constituency *i* over the land area in constituency *i* by combining data from the Office of National Statistics on the constituency size and NHS total population estimates; and we also add the share of a constituency who define their ethnicity as white, using data obtained from the House of Commons Library. Summary statistics are provided in Table S1.1 in the supplementary information. Finally, we include region fixed effects. Given the cross-sectional nature of the constituency data, we use robust standard errors.

### Individual level analysis

Our empirical analysis relies on a survey dataset compiled by YouGov, an organization that regularly fields surveys in a representative sample from a global online panel of 14 million people. YouGov has produced extensive surveys on the attitudes and behaviours of people in countries affected by COVID-19. These Covid-19 specific surveys have been used widely by both the academic community and policy makers.

We were granted access to a YouGov survey, consisting of 8485 individuals, fielded weekly to a nationally representative sample, over 5 weeks between the 4th of October and the 9th of November 2021. Each individual was asked the same series of survey questions, which include both information about their vote choice for a political party in the last general election in 2019 (for example Conservative, Labour, Green, Liberal Democrat or Brexit Party) and their vaccination status, as well as a series of relevant socio-demographic controls including their gender, age, the number of their children and their social class. Once all controls are included, the sample size for the individual-level regressions is 5967 observations.

This detailed individual-level information about vaccination allows us to create repeated cross-sections. We then estimate the following regression to model the probability of being vaccinated as a function of having voted for the Conservative party in the last election (or not) as well as relevant socio-demographic factors:

$${Vaccinated}_{jr}={\alpha }_{0}+ {\alpha }_{1}{Conservative}_{jr}+ \mathrm{\rm E}{Z}_{jr}+ {\gamma }_{r}+{\varepsilon }_{j}$$

The dependent variable is the vaccination status. It takes the value of one if the respondent received all the injections required to be fully vaccinated against Covid–19 or started the vaccination process (but need another shot), and zero otherwise. Using ordinary least squares (OLS) or logistic regressions does not change the results. The regressor of interest is whether or not the respondent voted for the Conservative party during the last general election in 2019. We also control for a set of relevant individual-level characteristics: how respondents voted during the Brexit referendum, respondents’ age and gender, whether they have children, and their socio-economic grade. The measures for Social Class are very well-established in past survey research and respondents can select whether they belong to one of six classes^71^.

Summary statistics are presented in Table S2.1 in the supplementary information. The vaccination rates for first and second doses approximate the NHS estimates around the time of the survey. For instance, nine in ten individuals aged 18 and over had been vaccinated with at least one dose according to official estimates recorded on 17th October 2021^72^. Our survey-based vaccination rate estimate is a slightly higher 92.4% but the survey is longer, ending in November so this higher vaccination rate is plausible, especially with mild measurement errors. Finally, we control for the location of each respondent by including region dummies and address potential heteroskedasticity by using robust standard errors.

### Robustness and sensitivity analysis

We discuss a number of robustness checks which are reported in the supplementary information. Table S1.2 shows that the sign and statistical significance of the Conservative vote share coefficient is not affected by the inclusion or exclusion of any one of the following controls: median house price, median wage, population density, the share of the population that is white, the share of population over 60, the median age, the index of material deprivations (IMD), the IMD for education, skills & training rank, nor the share of residents > 16 years old with no qualification. Table S1.3 replicates the analysis while including region fixed effects. At the individual level, including or excluding region fixed effects does not change the results (Table S2.5), nor does using OLS (model 2) as opposed to logistic regression (model 3). Table S1.8 replicates the results for vaccination rates in second doses while Table S1.9 does the same while including region fixed effects.

Next, Table S1.6 shows that the association between vaccination rates and the share of voters that voted neither for Labour nor for Conservative parties is not statistically significant across specifications. Including region fixed effects does not change this non-significant finding (Table S1.12). Distinguishing Labour and Conservative voter share in Tables S1.7 and S1.13 confirms that Conservative vote share is positively associated with vaccination (first or second doses), while the opposite is true for the Labour vote share.

Moreover, rerunning the analyses of vaccination rates for first or second doses by age group demonstrates a positive association between Conservative vote share and vaccination across nearly all age categories (Tables S1.14 and S1.15). At the individual level (Table S2.6), Conservative vote and vaccination are positively associated when restricting samples to middle-aged individuals (25 to 55) or older individuals (above 55), but not for those under 25 (although note that sample size is limited and few individuals under 25 vote for the Conservative party).

We further explore other sample restrictions by trichotomizing each control variable into three tertiles of their respective underlying distribution. We then rerunning our regression for each tertile of each control variable. For instance, we rerun our analysis while restricting the sample to the bottom third of age distribution, then the middle third, and then the top third of age distribution. We do this for every control variable. We run this sub-sample specific regressions for both first-dose (Table S1.16) and second-dose vaccination rates (Table S1.17). For first-dose vaccination rates, the association with Conservative vote share remains positive and statistically significant for all sub-samples created with the tertiles of the following variables: house prices; median wages; share of deprived Local Authorities; share with no education; share of white population; overall index of material deprivation (IMD); IMD of education rank. For two variables, there is no association when restricting the sample to their top tertile values: the coefficient for Conservative vote is not statistically significant when restricting the sample to include only individuals in the top tertile of share over 60 years old; and top tertile of the population median age. This result is intuitive because the vaccination rate among older people was so high that no partisan differences (or any other differences) could be discerned. Restricting survey analyses to different regions does not change results either (Table S2.7).

Some have argued that Brexit is a crucial additional political cleavage besides partisanship^73,74^. At the constituency level, Table S1.4 shows the share of the electorate that voted for Brexit in the 2016 EU referendum is negatively related to vaccination rates, while the Conservative vote share continues to be positively associated with vaccination. In Table S1.5 we replicate the results when region fixed effects are included, and the results are the same. Table S1.10 then demonstrates that using second-dose instead of first-dose vaccination rates does not change the results, nor does including region fixed effects (Table S1.11). At the individual level, having voted to leave the EU is clearly negatively and significantly associated with vaccination likelihood (Table S2.5)^75^.

Broader Covid-19 attitudes held by voter groups could in principle account for some of the small partisan differences in vaccination rates. Consistent with this attitudinal source of partisan differences, we find that Conservative voters are more likely to think that the government handled Covid-19 well (Table S2.8). By contrast, Conservative voters do not appear more likely to vaccinate because they take Covid-19 more seriously in general: they are less likely to declare improving hygiene or avoiding crowded places due to Covid-19, as well as less likely to wear masks or to avoid going to work than other voter groups (Table S2.9). Controlling for any one of these attitudes does not make the association between Conservative vote and vaccination status non-significant (Table S2.10).

## Supplementary Information

Supplementary Tables.

## Data Availability

All data and code used in the empirical analysis of the main text or the supplementary material are available upon request from Tim Vlandas at tim.vlandas@spi.ox.ac.uk.
