# Supplementary material for: Partisanship and Covid-19 vaccination in the UK
Source: Sci Rep. 2022 Nov 18;12:19785. doi: 10.1038/s41598-022-23035-w (PMC9672584; doi:10.1038/s41598-022-23035-w)
Supplement: Supplementary file 1 — Supplementary Tables. [file 41598_2022_23035_MOESM1_ESM.docx]

Supplementary Information for

**PARTISANSHIP AND COVID-19 VACCINATION IN THE UK**

*Scientific Reports (Nature Portfolio)*

**Margaryta Klymak^1^**, **Tim Vlandas^2*^**

1 University of Oxford.
Address: University of Oxford, Somerville College, Woodstock Rd, Oxford OX2 6HD, United Kingdom
Email: [margaryta.klymak@some.ox.ac.uk](mailto:margaryta.klymak@some.ox.ac.uk)

2 University of Oxford.
Address: University of Oxford, Department of Social Policy and Intervention, 32 Wellington Square, OX1 2ER Oxford, United Kingdom
Email: [tim.vlandas@spi.ox.ac.uk](mailto:tim.vlandas@spi.ox.ac.uk)

**Table of Contents**

[**S1. Constituency level data and analyses** 5](#_Toc113635660)

[*Table S1.1. Summary Statistics for constituency level data* 5](#_Toc113635661)

[*Table S1.2. Results for vaccination first doses with one control at a time* 7](#_Toc113635662)

[*Table S1.2. (cont.) Results for vaccination first doses with one control at a time* 8](#_Toc113635663)

[*Table S1.3. Results for vaccination first doses with one control at a time with region fixed effects* 9](#_Toc113635664)

[*Table S1.3. (cont.) Results for vaccination first doses with one control at a time with region fixed effects* 10](#_Toc113635665)

[*Table S1.4. Results for vaccination first doses with EU referendum share and one control at a time with region fixed effects* 11](#_Toc113635666)

[*Table S1.4. (cont.) Results for vaccination first doses with EU referendum share and one control at a time with region fixed effects* 12](#_Toc113635667)

[*Table S1.5. Results for vaccination rates (first doses) with all controls and region fixed effects* 13](#_Toc113635668)

[*Table S1.5. (cont.) Results for vaccination rates (first doses) with all controls and region fixed effects* 14](#_Toc113635669)

[*Table S1.6. Results for vaccination first doses regressed on votes share for non-Labour non-Conservative Parties* 1](#_Toc113635670)

[*Table S1.7. Results for vaccination first doses and controlling for Labour vote share* 1](#_Toc113635671)

[*Table S1.8. Results for vaccination second doses with one control at a time* 2](#_Toc113635672)

[*Table S1.8. (cont.) Results for vaccination second doses with one control at a time* 3](#_Toc113635673)

[*Table S1.9. Results for vaccination second doses with one control at a time and with region fixed effects* 4](#_Toc113635674)

[*Table S1.9. (cont.) Results for vaccination second doses with one control at a time and with region fixed effects* 5](#_Toc113635675)

[*Table S1.10. Results for vaccination second doses with EU referendum share and one control at a time with region fixed effects* 6](#_Toc113635676)

[*Table S1.10. (cont.) Results for vaccination second doses with EU referendum share and one control at a time with region fixed effects* 7](#_Toc113635677)

[*Table S1.11. Results for vaccination second doses with all controls and region fixed effects* 8](#_Toc113635678)

[*Table S1.11. (cont.) Results for vaccination second doses with all controls and region fixed effects* 9](#_Toc113635679)

[*Table S1.12. Results for vaccination second doses regressed on votes share for non-Labour non-Conservative Parties* 11](#_Toc113635680)

[*Table S1.13. Results for vaccination second doses and controlling for Labour vote share* 12](#_Toc113635681)

[*Table S1.14. Results for age-specific vaccination rates (first doses)* 1](#_Toc113635682)

[*Table S1.14. (cont.) Results for age-specific vaccination rates (first doses)* 3](#_Toc113635683)

[*Table S1.15. Results for age-specific vaccination rates (second doses)* 5](#_Toc113635684)

[*Table S1.15. (cont.) Results for age-specific vaccination rates (second doses)* 7](#_Toc113635685)

[*Table S1.16. Results for Conservative vote share and vaccination rate (first dose) - sample restrictions* 9](#_Toc113635686)

[*Table S1.16. (cont.) Conservative vote share and vaccination rate (first dose) - sample restrictions* 9](#_Toc113635687)

[*Table S1.16. (cont.) Results for Conservative vote share and vaccination rate (first dose) - sample restrictions* 10](#_Toc113635688)

[*Table S1.17. Results for Conservative vote share and vaccination rate (second dose) - sample restrictions* 10](#_Toc113635689)

[*Table S1.17. (cont.) Results for Conservative vote share and vaccination rate (second dose) - sample restrictions* 11](#_Toc113635690)

[*Table S1.17. (cont.) Results for Conservative vote share and vaccination rate (second dose) - sample restrictions* 11](#_Toc113635691)

[**S2. Individual level analyses** 1](#_Toc113635692)

[*Table S2.1. Summary Statistics YouGov survey* 1](#_Toc113635693)

[*Table S2.2. Past vote variable* 2](#_Toc113635694)

[*Table S2.3. Vaccination status* 3](#_Toc113635695)

[*Table S2.4. Regions* 4](#_Toc113635696)

[*Table S2.5. Results for vaccination status (at least one dose) and past conservative vote* 5](#_Toc113635697)

[*Table S2.6. Results for regressions of conservative vote share on vaccination status with age specific sub-samples* 7](#_Toc113635698)

[*Table S2.7. Results for vaccination status (at least one dose) and conservative vote - region sub-samples* 1](#_Toc113635699)

[*Table S2.7. (cont.) Results for vaccination status (at least one dose) and conservative vote - region sub-samples* 2](#_Toc113635700)

[*Table S2.8. Results for agreement that Government handled Covid-19 well regressed on Conservative vote* 3](#_Toc113635701)

[*Table S2.9. Results for different dependent variables regressed on Conservative vote* 5](#_Toc113635702)

[*Table S2.10. Results when adding more controls to regression on vaccination status (at least one dose)* 7](#_Toc113635703)

[*Table S2.11. Results for different operationalization of vaccination status* 1](#_Toc113635704)

# **S1. Constituency level data and analyses**

## *Table S1.1. Summary Statistics for constituency level data*

|  | **(1)** | **(2)** | **(3)** | **(4)** |
| --- | --- | --- | --- | --- |
| **Variables** | **N** | **Mean** | **SD** | **Source and notes** |
| First Dose | 532 | 66.86 | 7.716 | NHS vaccination data: <https://www.england.nhs.uk/statistics/statistical-work-areas/covid-19-vaccinations/> |
| Second Dose | 532 | 61.80 | 8.148 | NHS vaccination data: <https://www.england.nhs.uk/statistics/statistical-work-areas/covid-19-vaccinations/> |
| Median house price | 532 | 283,784 | 149,825 | House prices accessed at: <https://commonslibrary.parliament.uk/constituency-data-house-prices/>  For the purpose of regression analyses the data has been expressed in 1000s |
| Median wage | 463 | 24,831 | 4,388 | Office for National Statistics – Annual Survey of Hours and Earnings (ASHE) and Labour Force Survey (LFS). For the purpose of regression analyses the data has been expressed in 1000s |
| IMD rank | 532 | 266.9 | 154.1 | The Index of Multiple Deprivation (IMD) rank. This ranks every small area in England from most deprived area to least deprived area |
| IMD - Education, skills and training rank | 532 | 266.8 | 154.1 | The Index of Multiple Deprivation (IMD) for Education, skills and training rank. This index ranks every small area in England from most deprived area to least deprived area in terms of their performance on Education, skills and training |
| Population density | 532 | 277.8 | 365.6 | Office for National Statistics |
| Conservative vote | 532 | 46.67 | 15.76 | Electoral results available in Commons Online Library accessed at: <https://commonslibrary.parliament.uk/constituency-data-election-results/> |
| Labour | 532 | 34.81 | 17.51 | Electoral results available in Commons Online Library accessed at: <https://commonslibrary.parliament.uk/constituency-data-election-results/> |
| Liberal Democrats | 532 | 0.117 | 0.101 | Electoral results available in Commons Online Library accessed at: <https://commonslibrary.parliament.uk/constituency-data-election-results/> |
| Brexit | 532 | 0.0234 | 0.0415 | Electoral results available in Commons Online Library accessed at: <https://commonslibrary.parliament.uk/constituency-data-election-results/> |
| Green | 532 | 0.0293 | 0.0317 | Electoral results available in Commons Online Library accessed at: <https://commonslibrary.parliament.uk/constituency-data-election-results/> |
| Share of population over 60 | 532 | 0.239 | 0.0645 | Office for National Statistics |
| Median age in 2019 | 532 | 41.24 | 5.589 | Office for National Statistics |
| Share of vote for Leave in 2016 EU referendum | 570 | 53.50 | 10.84 | Commons Online Library accessed at: [https://commonslibrary.parliament.uk/](https://commonslibrary.parliament.uk/brexit-votes-by-constituency/) |
| Share of Lower-layer Super Output Areas in most deprived 100 | 532 | 9.976 | 14.04 | Official census and labour market statistics |
| Share of residents aged 16 and over with no qualification | 572 | 22.93 | 5.687 | Official census and labour market statistics |
| Share of population that self-identify as White | 532 | 86.54 | 15.74 | Ethnicity data available in Commons Online Library accessed at: <https://commonslibrary.parliament.uk/constituency-statistics-ethnicity/> |

##

## *Table S1.2. Results for vaccination first doses with one control at a time*

| **Model** | **1** | **2** | **3** | **4** | **5** |
| --- | --- | --- | --- | --- | --- |
| Conservative vote share | 0.327*** | 0.302*** | 0.161*** | 0.132*** | 0.0540*** |
|  | (0.0161) | (0.0177) | (0.0191) | (0.0141) | (0.0118) |
| Median House Price (in 1000s) | -0.0103*** |  |  |  |  |
|  | (0.00141) |  |  |  |  |
| Median wage (in 1000s) |  | -0.404*** |  |  |  |
|  |  | (0.0579) |  |  |  |
| Population density |  |  | -0.0113*** |  |  |
|  |  |  | (0.00105) |  |  |
| Share of population White |  |  |  | 0.339*** |  |
|  |  |  |  | (0.0183) |  |
| Share of population over 60 |  |  |  |  | 99.71*** |
|  |  |  |  |  | (3.125) |
| Constant | 54.51*** | 62.84*** | 62.48*** | 31.37*** | 40.53*** |
|  | (1.060) | (1.905) | (1.139) | (1.307) | (0.679) |
| Observations | 532 | 463 | 532 | 532 | 532 |
| R-squared | 0.507 | 0.509 | 0.630 | 0.774 | 0.833 |

*Note: Robust standard errors in parentheses; *** p<0.01, ** p<0.05, * p<0.1.*

## *Table S1.2. (cont.) Results for vaccination first doses with one control at a time*

| **Model** | **6** | **7** | **8** | **9** |
| --- | --- | --- | --- | --- |
| Conservative vote share | 0.0784*** | 0.270*** | 0.324*** | 0.335*** |
|  | (0.0131) | (0.0174) | (0.0161) | (0.0163) |
| Median age | 1.057*** |  |  |  |
|  | (0.0390) |  |  |  |
| IMD - Overall index rank |  | 0.0128*** |  |  |
|  |  | (0.00158) |  |  |
| IMD - Education, skills & training rank |  |  | 0.00803*** |  |
|  |  |  | (0.00168) |  |
| Share residents >16 years old with no qualification |  |  |  | -0.0692 |
|  |  |  |  | (0.0493) |
| Constant | 19.60*** | 50.88*** | 49.61*** | 52.80*** |
|  | (1.492) | (0.851) | (0.948) | (1.303) |
| Observations | 532 | 532 | 532 | 532 |
| R-squared | 0.780 | 0.515 | 0.493 | 0.470 |

*Note: Robust standard errors in parentheses; *** p<0.01, ** p<0.05, * p<0.1.*

## *Table S1.3. Results for vaccination first doses with one control at a time with region fixed effects*

| **Model** | **1** | **2** | **3** | **4** | **5** |
| --- | --- | --- | --- | --- | --- |
| Conservative vote share | 0.329*** | 0.322*** | 0.231*** | 0.157*** | 0.0645*** |
|  | (0.0165) | (0.0176) | (0.0214) | (0.0164) | (0.0154) |
| Median House Price | 0.00866** |  |  |  |  |
| *(in 1000s)* | (0.00386) |  |  |  |  |
| Median wage |  | -0.165*** |  |  |  |
| *(in 1000s)* |  | (0.0581) |  |  |  |
| Population density |  |  | -0.00769*** |  |  |
|  |  |  | (0.00127) |  |  |
| Share of population White |  |  |  | 0.314*** |  |
|  |  |  |  | (0.0250) |  |
| Share of population over 60 |  |  |  |  | 90.41*** |
|  |  |  |  |  | (3.835) |
| *Region fixed effects*  *(Ref: East of England)* |  |  |  |  |  |
| East Midlands | 2.497** | 1.005 | 1.433 | 1.367 | 0.672 |
|  | (1.159) | (1.132) | (0.987) | (0.888) | (0.669) |
| London | -5.842*** | -3.218*** | -0.236 | 1.235 | -2.013*** |
|  | (1.060) | (1.044) | (1.036) | (0.867) | (0.664) |
| North East | 9.527*** | 7.667*** | 6.553*** | 3.307*** | 1.794** |
|  | (1.317) | (1.196) | (1.053) | (0.899) | (0.709) |
| North West | 5.823*** | 4.835*** | 3.467*** | 1.378* | 0.143 |
|  | (1.086) | (0.984) | (0.916) | (0.727) | (0.623) |
| South East | 1.376* | 2.115** | 1.595** | 1.189** | 1.189** |
|  | (0.805) | (0.871) | (0.752) | (0.604) | (0.523) |
| South West | 5.257*** | 4.696*** | 4.575*** | 2.736*** | 0.925 |
|  | (0.804) | (0.861) | (0.728) | (0.628) | (0.619) |
| West Midlands | -0.344 | -1.526 | -0.757 | 0.270 | -1.540** |
|  | (1.034) | (1.034) | (0.890) | (0.691) | (0.599) |
| Yorkshire and the Humber | 5.158*** | 3.198*** | 2.863*** | 1.859** | 0.638 |
|  | (1.123) | (1.017) | (0.933) | (0.743) | (0.646) |
| Constant | 47.07*** | 54.12*** | 56.37*** | 30.99*** | 42.18*** |
|  | (1.769) | (1.956) | (1.540) | (1.855) | (0.954) |
| Observations | 532 | 463 | 532 | 532 | 532 |
| R-squared | 0.655 | 0.649 | 0.683 | 0.785 | 0.852 |

*Note: Robust standard errors in parentheses; *** p<0.01, ** p<0.05, * p<0.1.*

## *Table S1.3. (cont.) Results for vaccination first doses with one control at a time with region fixed effects*

| **Model** | **6** | **7** | **8** | **9** |
| --- | --- | --- | --- | --- |
| Conservative vote share | 0.0731*** | 0.231*** | 0.285*** | 0.328*** |
|  | (0.0153) | (0.0162) | (0.0148) | (0.0162) |
| Median age | 0.934*** |  |  |  |
|  | (0.0390) |  |  |  |
| IMD - Overall index rank |  | 0.0202*** |  |  |
|  |  | (0.00136) |  |  |
| IMD - Education, skills & training rank |  |  | 0.0172*** |  |
|  |  |  | (0.00123) |  |
| Share residents >16 years old with no qualification |  |  |  | -0.322*** |
|  |  |  |  | (0.0427) |
| *Region fixed effects (Ref: East of England)* |  |  |  |  |
| East Midlands | 1.027 | 2.141** | 1.813** | 2.181** |
|  | (0.704) | (0.911) | (0.916) | (1.030) |
| London | -4.573*** | -4.760*** | -7.393*** | -5.511*** |
|  | (0.714) | (0.812) | (0.855) | (0.887) |
| North East | 3.067*** | 9.932*** | 8.286*** | 9.282*** |
|  | (0.725) | (1.029) | (1.043) | (1.144) |
| North West | 0.481 | 5.945*** | 4.169*** | 5.318*** |
|  | (0.624) | (0.821) | (0.844) | (0.897) |
| South East | 1.044** | 0.649 | 0.309 | 0.576 |
|  | (0.521) | (0.715) | (0.737) | (0.773) |
| South West | 1.801*** | 4.752*** | 3.846*** | 4.276*** |
|  | (0.623) | (0.709) | (0.690) | (0.751) |
| West Midlands | -0.702 | 0.642 | -0.791 | 0.0216 |
|  | (0.620) | (0.818) | (0.813) | (0.896) |
| Yorkshire and the Humber | 1.327* | 5.127*** | 4.314*** | 4.967*** |
|  | (0.687) | (0.845) | (0.880) | (0.939) |
| Constant | 24.81*** | 48.59*** | 48.02*** | 57.15*** |
|  | (1.552) | (1.086) | (1.129) | (1.398) |
| Observations | 532 | 532 | 532 | 532 |
| R-squared | 0.842 | 0.744 | 0.735 | 0.683 |

*Note: Robust standard errors in parentheses; *** p<0.01, ** p<0.05, * p<0.1.*

## *Table S1.4. Results for vaccination first doses with EU referendum share and one control at a time with region fixed effects*

| **Model** | **1** | **2** | **3** | **4** | **5** |
| --- | --- | --- | --- | --- | --- |
| Conservative vote share | 0.408*** | 0.385*** | 0.296*** | 0.216*** | 0.125*** |
|  | (0.0237) | (0.0208) | (0.0229) | (0.0175) | (0.0167) |
| Leave vote in EU Referendum | -0.194*** | -0.178*** | -0.197*** | -0.137*** | -0.140*** |
|  | (0.0409) | (0.0278) | (0.0231) | (0.0209) | (0.0164) |
| Median House Price | -0.00299 |  |  |  |  |
| *(in 1000s)* | (0.00400) |  |  |  |  |
| Median wage |  | -0.225*** |  |  |  |
| *(in 1000s)* |  | (0.0600) |  |  |  |
| Population density |  |  | -0.00862*** |  |  |
|  |  |  | (0.00120) |  |  |
| Share of population White |  |  |  | 0.303*** |  |
|  |  |  |  | (0.0250) |  |
| Share of population over 60 |  |  |  |  | 88.40*** |
|  |  |  |  |  | (3.652) |
| *Region fixed effects*  *(Ref: East of England)* |  |  |  |  |  |
| East Midlands | 1.805* | 1.688 | 2.053** | 1.812** | 1.139* |
|  | (1.085) | (1.058) | (0.911) | (0.835) | (0.593) |
| London | -4.506*** | -4.190*** | -1.153 | 0.110 | -3.017*** |
|  | (0.991) | (0.922) | (0.912) | (0.826) | (0.610) |
| North East | 9.348*** | 9.075*** | 8.154*** | 4.722*** | 3.204*** |
|  | (1.241) | (1.209) | (1.058) | (0.903) | (0.709) |
| North West | 5.166*** | 5.499*** | 4.212*** | 2.117*** | 0.878 |
|  | (0.982) | (0.902) | (0.834) | (0.683) | (0.553) |
| South East | 1.167* | 1.618** | 0.875 | 0.718 | 0.701 |
|  | (0.708) | (0.751) | (0.668) | (0.554) | (0.429) |
| South West | 4.373*** | 4.263*** | 4.128*** | 2.529*** | 0.723 |
|  | (0.745) | (0.750) | (0.639) | (0.581) | (0.529) |
| West Midlands | -0.717 | -0.778 | 0.176 | 0.824 | -0.913* |
|  | (0.904) | (0.916) | (0.803) | (0.630) | (0.528) |
| Yorkshire and the Humber | 4.961*** | 4.461*** | 4.151*** | 2.925*** | 1.723*** |
|  | (1.020) | (0.949) | (0.873) | (0.697) | (0.593) |
| Constant | 57.25*** | 62.04*** | 63.95*** | 36.37*** | 47.17*** |
|  | (2.759) | (2.242) | (1.623) | (1.918) | (1.072) |
| Observations | 532 | 463 | 532 | 532 | 532 |
| R-squared | 0.676 | 0.682 | 0.722 | 0.804 | 0.872 |

*Note: Robust standard errors in parentheses; *** p<0.01, ** p<0.05, * p<0.1.*

## *Table S1.4. (cont.) Results for vaccination first doses with EU referendum share and one control at a time with region fixed effects*

| **Model** | **6** | **7** | **8** | **9** | **10** |
| --- | --- | --- | --- | --- | --- |
| Conservative vote share | 0.128*** | 0.178*** | 0.200*** | 0.232*** | 0.338*** |
|  | (0.0174) | (0.0266) | (0.0263) | (0.0253) | (0.0266) |
| Leave vote in EU Referendum | -0.118*** | 0.0867*** | 0.166*** | -0.0144 | -0.0254 |
|  | (0.0188) | (0.0333) | (0.0402) | (0.0292) | (0.0517) |
| Median age | 0.903*** |  |  |  |  |
|  | (0.0381) |  |  |  |  |
| IMD - Overall index rank |  | 0.0241*** |  |  |  |
|  |  | (0.00210) |  |  |  |
| IMD - Education, skills & training rank |  |  | 0.0247*** |  |  |
|  |  |  | (0.00228) |  |  |
| Share of deprived LSOAs |  |  |  | -0.233*** |  |
|  |  |  |  | (0.0225) |  |
| Share residents >16 years old with no qualification |  |  |  |  | -0.286*** |
|  |  |  |  |  | (0.0863) |
| *Region fixed effects*  *(Ref: East of England)* |  |  |  |  |  |
| East Midlands | 1.421** | 1.983** | 1.412 | 2.255** | 2.188** |
|  | (0.647) | (0.912) | (0.918) | (0.975) | (1.025) |
| London | -5.358*** | -4.338*** | -7.806*** | -6.895*** | -5.499*** |
|  | (0.704) | (0.821) | (0.902) | (0.925) | (0.886) |
| North East | 4.305*** | 9.503*** | 6.871*** | 9.885*** | 9.378*** |
|  | (0.734) | (1.025) | (1.101) | (1.036) | (1.157) |
| North West | 1.157** | 5.797*** | 3.194*** | 6.784*** | 5.361*** |
|  | (0.583) | (0.835) | (0.921) | (0.853) | (0.898) |
| South East | 0.647 | 0.749 | 0.274 | 1.209 | 0.616 |
|  | (0.455) | (0.738) | (0.792) | (0.777) | (0.768) |
| South West | 1.663*** | 4.904*** | 3.726*** | 4.656*** | 4.293*** |
|  | (0.547) | (0.736) | (0.739) | (0.743) | (0.743) |
| West Midlands | -0.200 | 0.626 | -1.319 | 1.034 | -0.0105 |
|  | (0.567) | (0.840) | (0.871) | (0.843) | (0.891) |
| Yorkshire and the Humber | 2.266*** | 4.725*** | 3.270*** | 6.042*** | 5.038*** |
|  | (0.669) | (0.847) | (0.919) | (0.894) | (0.942) |
| Constant | 29.71*** | 45.39*** | 41.59*** | 56.98*** | 57.18*** |
|  | (1.722) | (1.642) | (1.885) | (1.242) | (1.397) |
| Observations | 532 | 532 | 532 | 532 | 532 |
| R-squared | 0.856 | 0.748 | 0.746 | 0.747 | 0.683 |

*Note: Robust standard errors in parentheses; *** p<0.01, ** p<0.05, * p<0.1.*

## *Table S1.5. Results for vaccination rates (first doses) with all controls and region fixed effects*

| **Model** | **1** | **2** | **3** | **4** | **5** |
| --- | --- | --- | --- | --- | --- |
| Conservative vote share | 0.379*** | 0.281*** | 0.228*** | 0.179*** | 0.0847*** |
|  | (0.0256) | (0.0296) | (0.0198) | (0.0224) | (0.0165) |
| Leave vote in EU Referendum | -0.165*** | -0.160*** | -0.181*** | -0.177*** | -0.169*** |
|  | (0.0464) | (0.0383) | (0.0327) | (0.0288) | (0.0217) |
| Median House Price | 0.00190 | 0.00433 | -0.00470 | -0.00278 | -0.00656*** |
| *(in 1000s)* | (0.00508) | (0.00386) | (0.00369) | (0.00310) | (0.00245) |
| Median wage | -0.232*** | -0.145* | -0.129** | -0.0837 | 0.120*** |
| *(in 1000s)* | (0.0647) | (0.0744) | (0.0568) | (0.0623) | (0.0418) |
| Population density |  | -0.00790*** |  | -0.00475*** | -0.00101 |
|  |  | (0.00157) |  | (0.00119) | (0.000832) |
| Share of population White |  |  | 0.304*** | 0.283*** | 0.175*** |
|  |  |  | (0.0297) | (0.0295) | (0.0231) |
| Share of population over 60 |  |  |  |  | 73.32*** |
|  |  |  |  |  | (4.075) |
| *Region fixed effects*  *(Ref: East of England)* |  |  |  |  |  |
| East Midlands | 1.846 | 2.309** | 1.158 | 1.484 | 0.300 |
|  | (1.162) | (1.046) | (0.960) | (0.917) | (0.639) |
| London | -4.513*** | -1.325 | 1.382 | 2.899*** | 0.793 |
|  | (1.104) | (1.084) | (1.068) | (1.112) | (0.813) |
| North East | 9.248*** | 8.535*** | 4.047*** | 3.971*** | 0.964 |
|  | (1.309) | (1.186) | (1.068) | (0.997) | (0.678) |
| North West | 5.667*** | 5.026*** | 1.700** | 1.584** | -0.815 |
|  | (1.032) | (0.951) | (0.835) | (0.791) | (0.552) |
| South East | 1.597** | 1.285* | 1.100* | 0.946* | 0.245 |
|  | (0.757) | (0.723) | (0.584) | (0.571) | (0.397) |
| South West | 4.358*** | 4.463*** | 2.165*** | 2.378*** | -0.230 |
|  | (0.802) | (0.727) | (0.664) | (0.622) | (0.465) |
| West Midlands | -0.648 | 0.391 | 0.482 | 1.031 | -0.536 |
|  | (0.980) | (0.918) | (0.708) | (0.690) | (0.471) |
| Yorkshire and the Humber | 4.606*** | 4.245*** | 2.198*** | 2.145*** | 0.302 |
|  | (1.049) | (0.971) | (0.795) | (0.756) | (0.553) |
| Constant | 61.20*** | 64.45*** | 42.62*** | 45.83*** | 38.38*** |
|  | (3.233) | (3.203) | (3.167) | (3.292) | (2.367) |
| Observations | 463 | 463 | 463 | 463 | 463 |
| R-squared | 0.682 | 0.715 | 0.807 | 0.818 | 0.908 |

*Note: Robust standard errors in parentheses; *** p<0.01, ** p<0.05, * p<0.1.*

## *Table S1.5. (cont.) Results for vaccination rates (first doses) with all controls and region fixed effects*

| **Model** | **6** | **7** | **8** | **9** | **10** |
| --- | --- | --- | --- | --- | --- |
| Conservative vote share | 0.0798*** | 0.0803*** | 0.0900*** | 0.0750*** | 0.158*** |
|  | (0.0161) | (0.0221) | (0.0216) | (0.0198) | (0.0279) |
| Leave vote in EU Referendum | -0.180*** | -0.00498 | 0.0446 | -0.0589** | -0.113** |
|  | (0.0198) | (0.0321) | (0.0318) | (0.0272) | (0.0501) |
| Median House Price | -0.0103*** | -0.00836*** | -0.0107*** | -0.00311 | -0.00341 |
| *(in 1000s)* | (0.00211) | (0.00220) | (0.00227) | (0.00227) | (0.00314) |
| Median wage | 0.0667* | -0.147*** | -0.101* | -0.107** | -0.102 |
| *(in 1000s)* | (0.0362) | (0.0536) | (0.0533) | (0.0513) | (0.0636) |
| Population density | -0.00110 | -6.37e-05 | -0.000972 | -0.00296*** | -0.00430*** |
|  | (0.000781) | (0.00117) | (0.00104) | (0.000969) | (0.00120) |
| Share of population White | 0.202*** | 0.268*** | 0.273*** | 0.271*** | 0.282*** |
|  | (0.0237) | (0.0267) | (0.0279) | (0.0248) | (0.0296) |
| Median age | 0.738*** |  |  |  |  |
|  | (0.0389) |  |  |  |  |
| IMD - Overall index rank |  | 0.0196*** |  |  |  |
|  |  | (0.00213) |  |  |  |
| IMD - Education, skills & training rank |  |  | 0.0203*** |  |  |
|  |  |  | (0.00192) |  |  |
| Share of deprived LSOAs |  |  |  | -0.185*** |  |
|  |  |  |  | (0.0174) |  |
| Share residents >16 years old with no qualification |  |  |  |  | -0.136 |
|  |  |  |  |  | (0.0850) |
| *Region fixed effects*  *(Ref: East of England)* |  |  |  |  |  |
| East Midlands | 0.240 | 1.104 | 0.384 | 1.742** | 1.531* |
|  | (0.694) | (0.818) | (0.853) | (0.846) | (0.923) |
| London | 0.0818 | 2.298** | 0.152 | 0.369 | 2.631** |
|  | (0.792) | (1.025) | (0.985) | (0.999) | (1.088) |
| North East | 0.990 | 4.307*** | 1.713* | 4.660*** | 3.902*** |
|  | (0.683) | (0.921) | (0.960) | (0.868) | (1.009) |
| North West | -1.291** | 2.090*** | -0.409 | 3.045*** | 1.574* |
|  | (0.571) | (0.702) | (0.777) | (0.745) | (0.801) |
| South East | 0.325 | 1.051** | 0.607 | 1.137* | 0.809 |
|  | (0.397) | (0.533) | (0.575) | (0.591) | (0.581) |
| South West | 0.192 | 2.636*** | 1.412** | 2.637*** | 2.252*** |
|  | (0.488) | (0.597) | (0.611) | (0.629) | (0.643) |
| West Midlands | -0.0978 | 1.221** | -0.466 | 1.989*** | 1.212* |
|  | (0.481) | (0.618) | (0.644) | (0.659) | (0.715) |
| Yorkshire and the Humber | 0.101 | 2.001*** | 0.476 | 3.240*** | 2.119*** |
|  | (0.566) | (0.662) | (0.718) | (0.687) | (0.760) |
| Constant | 26.40*** | 39.18*** | 36.62*** | 47.23*** | 47.18*** |
|  | (2.413) | (2.921) | (3.193) | (2.753) | (3.302) |
| Observations | 463 | 463 | 463 | 463 | 463 |
| R-squared | 0.910 | 0.850 | 0.855 | 0.861 | 0.820 |

*Note: Robust standard errors in parentheses; *** p<0.01, ** p<0.05, * p<0.1. Certain variables cannot be included together as they are too correlated which would result in multicollinearity (e.g. age variables and IMD, IMD, deprivation and low education).*

## *Table S1.6. Results for vaccination first doses regressed on votes share for non-Labour non-Conservative Parties*

| **Model** | **1** | **2** | **3** | **4** | **5** | **6** | **7** |
| --- | --- | --- | --- | --- | --- | --- | --- |
| Vote share for neither Conservative nor Labour | 0.0873*** | 0.0481** | 0.0242 | 0.0179 | -0.0138 | -0.0202 | 0.00160 |
|  | (0.0320) | (0.0228) | (0.0229) | (0.0197) | (0.0165) | (0.0166) | (0.0187) |
| Median House Price | 0.0135** | 0.0161*** | 0.00511 | 0.00818** | -0.00822*** | -0.0102*** | 0.000246 |
| *(in 1000s)* | (0.00527) | (0.00338) | (0.00414) | (0.00331) | (0.00224) | (0.00240) | (0.00331) |
| Median wage | -0.480*** | -0.161* | -0.181*** | -0.0849 | -0.150*** | -0.0963 | -0.118 |
| *(in 1000s)* | (0.0882) | (0.0963) | (0.0675) | (0.0743) | (0.0572) | (0.0604) | (0.0716) |
| Population density |  | -0.0155*** |  | -0.00771*** | -0.00165 | -0.00403*** | -0.00695*** |
|  |  | (0.00150) |  | (0.00120) | (0.00114) | (0.00105) | (0.00118) |
| Share of population White |  |  | 0.404*** | 0.320*** | 0.298*** | 0.318*** | 0.325*** |
|  |  |  | (0.0272) | (0.0288) | (0.0255) | (0.0278) | (0.0302) |
| IMD - Overall index rank |  |  |  |  | 0.0212*** |  |  |
|  |  |  |  |  | (0.00165) |  |  |
| IMD - Education, skills & training rank |  |  |  |  |  | 0.0188*** |  |
|  |  |  |  |  |  | (0.00145) |  |
| Share residents >16 years old with no qualification |  |  |  |  |  |  | -0.262*** |
|  |  |  |  |  |  |  | (0.0529) |
| Constant | 74.21*** | 68.04*** | 33.95*** | 39.29*** | 40.61*** | 40.87*** | 48.09*** |
|  | (2.200) | (2.414) | (3.331) | (3.563) | (2.923) | (3.164) | (3.650) |
| *Region fixed effects* | *YES* | *YES* | *YES* | *YES* | *YES* | *YES* | *YES* |
| Observations | 463 | 463 | 463 | 463 | 463 | 463 | 463 |
| R-squared | 0.408 | 0.630 | 0.744 | 0.785 | 0.842 | 0.839 | 0.799 |

*Note: Robust standard errors in parentheses; *** p<0.01, ** p<0.05, * p<0.1.*

## *Table S1.7. Results for vaccination first doses and controlling for Labour vote share*

| **Model** | **1** | **2** | **3** | **4** |
| --- | --- | --- | --- | --- |
| Conservative vote share | 0.0535** | 0.0518*** | 0.0757*** | 0.0672*** |
|  | (0.0220) | (0.0193) | (0.0188) | (0.0208) |
| Labour party vote share | -0.100*** | -0.0504*** | -0.0585*** | -0.0908*** |
|  | (0.0210) | (0.0186) | (0.0180) | (0.0201) |
| Median House Price | 0.00341 | -0.00976*** | -0.0134*** | -0.00512 |
| *(in 1000s)* | (0.00336) | (0.00217) | (0.00220) | (0.00311) |
| Median wage | -0.0882 | -0.145*** | -0.0982* | -0.122** |
| *(in 1000s)* | (0.0630) | (0.0515) | (0.0512) | (0.0594) |
| Population density | -0.00352*** | 0.000449 | -0.000621 | -0.00263** |
|  | (0.00129) | (0.00113) | (0.00104) | (0.00122) |
| Share of population White | 0.255*** | 0.256*** | 0.261*** | 0.258*** |
|  | (0.0285) | (0.0270) | (0.0286) | (0.0300) |
| IMD - Overall index rank |  | 0.0189*** |  |  |
|  |  | (0.00158) |  |  |
| IMD - Education, skills & training rank |  |  | 0.0177*** |  |
|  |  |  | (0.00138) |  |
| Share residents >16 years old with no qualification |  |  |  | -0.271*** |
|  |  |  |  | (0.0473) |
| *Region fixed effects*  *(Ref: East of England)* |  |  |  |  |
| East Midlands | 1.958** | 1.127 | 0.447 | 1.681* |
|  | (0.954) | (0.811) | (0.844) | (0.933) |
| London | 1.720 | 2.292** | 0.687 | 2.097* |
|  | (1.179) | (1.031) | (0.965) | (1.095) |
| North East | 4.185*** | 4.295*** | 1.962** | 3.876*** |
|  | (1.070) | (0.911) | (0.953) | (1.016) |
| North West | 2.575*** | 2.299*** | 0.00785 | 2.078** |
|  | (0.873) | (0.704) | (0.784) | (0.830) |
| South East | 0.820 | 0.914* | 0.468 | 0.458 |
|  | (0.600) | (0.530) | (0.563) | (0.588) |
| South West | 2.641*** | 2.402*** | 1.138* | 1.860*** |
|  | (0.642) | (0.567) | (0.593) | (0.651) |
| West Midlands | 1.196 | 1.159* | -0.390 | 1.347* |
|  | (0.760) | (0.614) | (0.627) | (0.704) |
| Yorkshire and the Humber | 2.123** | 1.938*** | 0.588 | 1.991*** |
|  | (0.867) | (0.660) | (0.703) | (0.769) |
| Constant | 46.10*** | 43.42*** | 43.90*** | 54.23*** |
| Observations | 463 | 463 | 463 | 463 |
| R-squared | 0.809 | 0.852 | 0.857 | 0.824 |

*Note: Robust standard errors in parentheses; *** p<0.01, ** p<0.05, * p<0.1.*

## *Table S1.8. Results for vaccination second doses with one control at a time*

| **Model** | **1** | **2** | **3** | **4** | **5** |
| --- | --- | --- | --- | --- | --- |
| Conservative vote share | 0.349*** | 0.323*** | 0.180*** | 0.144*** | 0.0595*** |
|  | (0.0170) | (0.0186) | (0.0203) | (0.0147) | (0.0122) |
| Median House Price | -0.00972*** |  |  |  |  |
| *(in 1000s)* | (0.00150) |  |  |  |  |
| Median wage |  | -0.421*** |  |  |  |
| *(in 1000s)* |  | (0.0604) |  |  |  |
| Population density |  |  | -0.0115*** |  |  |
|  |  |  | (0.00111) |  |  |
| Share of population White |  |  |  | 0.354*** |  |
|  |  |  |  | (0.0188) |  |
| Share of population over 60 |  |  |  |  | 105.4*** |
|  |  |  |  |  | (3.221) |
| Constant | 48.27*** | 57.22*** | 56.57*** | 24.42*** | 33.86*** |
|  | (1.130) | (1.999) | (1.215) | (1.335) | (0.710) |
| Observations | 532 | 463 | 532 | 532 | 532 |
| R-squared | 0.507 | 0.516 | 0.625 | 0.775 | 0.841 |

*Note: Robust standard errors in parentheses; *** p<0.01, ** p<0.05, * p<0.1.*

## *Table S1.8. (cont.) Results for vaccination second doses with one control at a time*

| **Model** | **6** | **7** | **8** | **9** | **10** |
| --- | --- | --- | --- | --- | --- |
| Conservative vote share | 0.0821*** | 0.284*** | 0.343*** | 0.322*** | 0.357*** |
|  | (0.0131) | (0.0180) | (0.0167) | (0.0188) | (0.0171) |
| Median age | 1.131*** |  |  |  |  |
|  | (0.0398) |  |  |  |  |
| IMD - Overall index rank |  | 0.0141*** |  |  |  |
|  |  | (0.00164) |  |  |  |
| IMD - Education, skills & training rank |  |  | 0.00934*** |  |  |
|  |  |  | (0.00174) |  |  |
| Share of deprived LSOAs |  |  |  | -0.0858*** |  |
|  |  |  |  | (0.0222) |  |
| Share residents >16 years old with no qualification |  |  |  |  | -0.0904* |
|  |  |  |  |  | (0.0513) |
| Constant | 11.34*** | 44.77*** | 43.28*** | 47.63*** | 47.21*** |
|  | (1.531) | (0.886) | (0.994) | (1.090) | (1.334) |
| Observations | 532 | 532 | 532 | 532 | 532 |
| R-squared | 0.795 | 0.527 | 0.506 | 0.493 | 0.479 |

*Note: Robust standard errors in parentheses; *** p<0.01, ** p<0.05, * p<0.1.*

## *Table S1.9. Results for vaccination second doses with one control at a time and with region fixed effects*

| **Model** | **1** | **2** | **3** | **4** | **5** |
| --- | --- | --- | --- | --- | --- |
| Conservative vote share | 0.354*** | 0.347*** | 0.251*** | 0.168*** | 0.0694*** |
|  | (0.0176) | (0.0187) | (0.0228) | (0.0172) | (0.0162) |
| Median House Price | 0.00977** |  |  |  |  |
| *(in 1000s)* | (0.00420) |  |  |  |  |
| Median wage |  | -0.181*** |  |  |  |
| *(in 1000s)* |  | (0.0607) |  |  |  |
| Population density |  |  | -0.00809*** |  |  |
|  |  |  | (0.00134) |  |  |
| Share of population White |  |  |  | 0.341*** |  |
|  |  |  |  | (0.0261) |  |
| Share of population over 60 |  |  |  |  | 97.39*** |
|  |  |  |  |  | (4.009) |
| *Region fixed effects*  *(Ref: East of England)* |  |  |  |  |  |
| East Midlands | 2.744** | 1.092 | 1.551 | 1.476 | 0.728 |
|  | (1.217) | (1.175) | (1.027) | (0.918) | (0.672) |
| London | -5.615*** | -2.717** | 0.434 | 2.142** | -1.390** |
|  | (1.123) | (1.105) | (1.093) | (0.905) | (0.688) |
| North East | 10.23*** | 8.090*** | 6.995*** | 3.429*** | 1.830** |
|  | (1.418) | (1.275) | (1.128) | (0.961) | (0.744) |
| North West | 6.190*** | 5.034*** | 3.625*** | 1.323* | 0.0145 |
|  | (1.164) | (1.042) | (0.978) | (0.763) | (0.648) |
| South East | 1.481* | 2.280** | 1.739** | 1.295** | 1.298** |
|  | (0.851) | (0.917) | (0.792) | (0.629) | (0.537) |
| South West | 5.973*** | 5.337*** | 5.227*** | 3.224*** | 1.287** |
|  | (0.855) | (0.910) | (0.770) | (0.659) | (0.638) |
| West Midlands | -0.194 | -1.521 | -0.697 | 0.432 | -1.528** |
|  | (1.100) | (1.090) | (0.944) | (0.723) | (0.625) |
| Yorkshire and the Humber | 5.758*** | 3.576*** | 3.253*** | 2.129*** | 0.829 |
|  | (1.199) | (1.078) | (0.991) | (0.777) | (0.670) |
| Constant | 40.21*** | 48.06*** | 50.19*** | 22.89*** | 35.08*** |
|  | (1.906) | (2.058) | (1.637) | (1.936) | (0.992) |
| Observations | 532 | 463 | 532 | 532 | 532 |
| R-squared | 0.652 | 0.647 | 0.679 | 0.788 | 0.856 |

*Note: Robust standard errors in parentheses; *** p<0.01, ** p<0.05, * p<0.1.*

## *Table S1.9. (cont.) Results for vaccination second doses with one control at a time and with region fixed effects*

| **Model** | **6** | **7** | **8** | **9** | **10** |
| --- | --- | --- | --- | --- | --- |
| Conservative vote share | 0.0777*** | 0.250*** | 0.307*** | 0.242*** | 0.353*** |
|  | (0.0161) | (0.0172) | (0.0158) | (0.0173) | (0.0173) |
| Median age | 1.010*** |  |  |  |  |
|  | (0.0402) |  |  |  |  |
| IMD - Overall index rank |  | 0.0217*** |  |  |  |
|  |  | (0.00142) |  |  |  |
| IMD - Education, skills & training rank |  |  | 0.0186*** |  |  |
|  |  |  | (0.00128) |  |  |
| Share of deprived LSOAs |  |  |  | -0.256*** |  |
|  |  |  |  | (0.0189) |  |
| Share residents >16 years old with no qualification |  |  |  |  | -0.345*** |
|  |  |  |  |  | (0.0451) |
| *Region fixed effects*  *(Ref: East of England)* |  |  |  |  |  |
| East Midlands | 1.109 | 2.309** | 1.959** | 2.399** | 2.350** |
|  | (0.711) | (0.951) | (0.957) | (1.013) | (1.077) |
| London | -4.150*** | -4.346*** | -7.196*** | -6.605*** | -5.147*** |
|  | (0.745) | (0.860) | (0.903) | (0.994) | (0.937) |
| North East | 3.183*** | 10.59*** | 8.825*** | 10.44*** | 9.890*** |
|  | (0.761) | (1.094) | (1.110) | (1.040) | (1.221) |
| North West | 0.362 | 6.261*** | 4.350*** | 7.141*** | 5.585*** |
|  | (0.650) | (0.878) | (0.903) | (0.905) | (0.959) |
| South East | 1.139** | 0.720 | 0.346 | 1.364 | 0.645 |
|  | (0.535) | (0.755) | (0.780) | (0.830) | (0.816) |
| South West | 2.219*** | 5.411*** | 4.431*** | 5.334*** | 4.901*** |
|  | (0.643) | (0.754) | (0.736) | (0.799) | (0.800) |
| West Midlands | -0.623 | 0.816 | -0.720 | 1.223 | 0.146 |
|  | (0.647) | (0.866) | (0.862) | (0.890) | (0.949) |
| Yorkshire and the Humber | 1.560** | 5.660*** | 4.790*** | 6.579*** | 5.484*** |
|  | (0.713) | (0.895) | (0.934) | (0.929) | (0.998) |
| Constant | 16.27*** | 41.98*** | 41.35*** | 50.62*** | 51.15*** |
|  | (1.604) | (1.153) | (1.200) | (1.300) | (1.474) |
| Observations | 532 | 532 | 532 | 532 | 532 |
| R-squared | 0.847 | 0.743 | 0.735 | 0.746 | 0.680 |

*Note: Robust standard errors in parentheses; *** p<0.01, ** p<0.05, * p<0.1.*

## *Table S1.10. Results for vaccination second doses with EU referendum share and one control at a time with region fixed effects*

| **Model** | **1** | **2** | **3** | **4** | **5** |
| --- | --- | --- | --- | --- | --- |
| Conservative vote share | 0.441*** | 0.416*** | 0.323*** | 0.234*** | 0.137*** |
|  | (0.0255) | (0.0223) | (0.0246) | (0.0186) | (0.0177) |
| Leave vote in EU Referendum | -0.213*** | -0.197*** | -0.217*** | -0.154*** | -0.157*** |
|  | (0.0439) | (0.0293) | (0.0244) | (0.0218) | (0.0170) |
| Median House Price | -0.00306 |  |  |  |  |
| *(in 1000s)* | (0.00433) |  |  |  |  |
| Median wage |  | -0.248*** |  |  |  |
| *(in 1000s)* |  | (0.0628) |  |  |  |
| Population density |  |  | -0.00911*** |  |  |
|  |  |  | (0.00124) |  |  |
| Share of population White |  |  |  | 0.328*** |  |
|  |  |  |  | (0.0262) |  |
| Share of population over 60 |  |  |  |  | 95.15*** |
|  |  |  |  |  | (3.800) |
| *Region fixed effects*  *(Ref: East of England)* |  |  |  |  |  |
| East Midlands | 1.982* | 1.849* | 2.235** | 1.973** | 1.250** |
|  | (1.132) | (1.097) | (0.945) | (0.861) | (0.587) |
| London | -4.145*** | -3.793*** | -0.579 | 0.884 | -2.513*** |
|  | (1.045) | (0.962) | (0.952) | (0.856) | (0.618) |
| North East | 10.04*** | 9.650*** | 8.764*** | 5.011*** | 3.408*** |
|  | (1.324) | (1.285) | (1.127) | (0.960) | (0.735) |
| North West | 5.467*** | 5.770*** | 4.448*** | 2.149*** | 0.837 |
|  | (1.045) | (0.949) | (0.885) | (0.712) | (0.564) |
| South East | 1.251* | 1.730** | 0.944 | 0.768 | 0.752* |
|  | (0.741) | (0.783) | (0.698) | (0.573) | (0.428) |
| South West | 4.999*** | 4.857*** | 4.734*** | 2.993*** | 1.062** |
|  | (0.788) | (0.787) | (0.672) | (0.607) | (0.536) |
| West Midlands | -0.604 | -0.694 | 0.333 | 1.051 | -0.827 |
|  | (0.954) | (0.960) | (0.847) | (0.654) | (0.545) |
| Yorkshire and the Humber | 5.540*** | 4.975*** | 4.675*** | 3.322*** | 2.042*** |
|  | (1.083) | (1.005) | (0.927) | (0.726) | (0.611) |
| Constant | 51.41*** | 56.83*** | 58.56*** | 28.91*** | 40.65*** |
|  | (2.960) | (2.341) | (1.688) | (2.005) | (1.105) |
| Observations | 532 | 463 | 532 | 532 | 532 |
| R-squared | 0.675 | 0.683 | 0.721 | 0.810 | 0.878 |

*Note: Robust standard errors in parentheses; *** p<0.01, ** p<0.05, * p<0.1.*

## *Table S1.10. (cont.) Results for vaccination second doses with EU referendum share and one control at a time with region fixed effects*

| **Model** | **6** | **7** | **8** | **9** | **10** |
| --- | --- | --- | --- | --- | --- |
| Conservative vote share | 0.139*** | 0.200*** | 0.222*** | 0.256*** | 0.375*** |
|  | (0.0185) | (0.0287) | (0.0284) | (0.0271) | (0.0285) |
| Leave vote in EU Referendum | -0.132*** | 0.0809** | 0.165*** | -0.0242 | -0.0530 |
|  | (0.0196) | (0.0358) | (0.0431) | (0.0311) | (0.0553) |
| Median age | 0.974*** |  |  |  |  |
|  | (0.0391) |  |  |  |  |
| IMD - Overall index rank |  | 0.0253*** |  |  |  |
|  |  | (0.00222) |  |  |  |
| IMD - Education, skills & training rank |  |  | 0.0261*** |  |  |
|  |  |  | (0.00241) |  |  |
| Share of deprived LSOAs |  |  |  | -0.247*** |  |
|  |  |  |  | (0.0239) |  |
| Share residents >16 years old with no qualification |  |  |  |  | -0.269*** |
|  |  |  |  |  | (0.0917) |
| *Region fixed effects*  *(Ref: East of England)* |  |  |  |  |  |
| East Midlands | 1.552** | 2.162** | 1.558 | 2.449** | 2.364** |
|  | (0.646) | (0.951) | (0.961) | (1.015) | (1.065) |
| London | -5.034*** | -3.953*** | -7.608*** | -6.654*** | -5.122*** |
|  | (0.730) | (0.866) | (0.958) | (0.972) | (0.930) |
| North East | 4.575*** | 10.19*** | 7.415*** | 10.59*** | 10.09*** |
|  | (0.762) | (1.097) | (1.182) | (1.098) | (1.235) |
| North West | 1.122* | 6.122*** | 3.379*** | 7.170*** | 5.675*** |
|  | (0.599) | (0.892) | (0.984) | (0.904) | (0.953) |
| South East | 0.693 | 0.813 | 0.311 | 1.297 | 0.729 |
|  | (0.458) | (0.778) | (0.836) | (0.815) | (0.802) |
| South West | 2.064*** | 5.552*** | 4.312*** | 5.293*** | 4.938*** |
|  | (0.557) | (0.779) | (0.787) | (0.783) | (0.782) |
| West Midlands | -0.0582 | 0.801 | -1.246 | 1.242 | 0.0787 |
|  | (0.588) | (0.887) | (0.922) | (0.884) | (0.935) |
| Yorkshire and the Humber | 2.617*** | 5.285*** | 3.749*** | 6.676*** | 5.633*** |
|  | (0.690) | (0.901) | (0.978) | (0.949) | (0.999) |
| Constant | 21.77*** | 39.00*** | 34.95*** | 51.18*** | 51.22*** |
|  | (1.771) | (1.735) | (1.990) | (1.297) | (1.470) |
| Observations | 532 | 532 | 532 | 532 | 532 |
| R-squared | 0.863 | 0.747 | 0.745 | 0.747 | 0.681 |

*Note: Robust standard errors in parentheses; *** p<0.01, ** p<0.05, * p<0.1.*

## *Table S1.11. Results for vaccination second doses with all controls and region fixed effects*

| **Model** | **1** | **2** | **3** | **4** | **5** |
| --- | --- | --- | --- | --- | --- |
| Conservative vote share | 0.409*** | 0.305*** | 0.245*** | 0.195*** | 0.0940*** |
|  | (0.0275) | (0.0316) | (0.0209) | (0.0232) | (0.0167) |
| Leave vote in EU Referendum | -0.181*** | -0.176*** | -0.199*** | -0.194*** | -0.186*** |
|  | (0.0496) | (0.0412) | (0.0346) | (0.0306) | (0.0229) |
| Median House Price | 0.00228 | 0.00486 | -0.00485 | -0.00286 | -0.00689** |
| *(in 1000s)* | (0.00544) | (0.00417) | (0.00396) | (0.00336) | (0.00268) |
| Median wage | -0.257*** | -0.164** | -0.145** | -0.0979 | 0.120*** |
| *(in 1000s)* | (0.0676) | (0.0782) | (0.0590) | (0.0649) | (0.0424) |
| Population density |  | -0.00836*** |  | -0.00494*** | -0.000940 |
|  |  | (0.00164) |  | (0.00121) | (0.000811) |
| Share of population White |  |  | 0.329*** | 0.307*** | 0.192*** |
|  |  |  | (0.0312) | (0.0309) | (0.0237) |
| Share of population over 60 |  |  |  |  | 78.35*** |
|  |  |  |  |  | (4.066) |
| *Region fixed effects*  *(Ref: East of England)* |  |  |  |  |  |
| East Midlands | 2.039* | 2.530** | 1.295 | 1.634* | 0.369 |
|  | (1.212) | (1.091) | (0.995) | (0.952) | (0.635) |
| London | -4.182*** | -0.809 | 2.195** | 3.773*** | 1.523* |
|  | (1.159) | (1.141) | (1.107) | (1.156) | (0.830) |
| North East | 9.858*** | 9.104*** | 4.232*** | 4.153*** | 0.940 |
|  | (1.394) | (1.264) | (1.131) | (1.056) | (0.699) |
| North West | 5.972*** | 5.294*** | 1.681* | 1.560* | -1.003* |
|  | (1.094) | (1.008) | (0.870) | (0.825) | (0.550) |
| South East | 1.704** | 1.374* | 1.167* | 1.007* | 0.258 |
|  | (0.791) | (0.754) | (0.599) | (0.586) | (0.380) |
| South West | 4.972*** | 5.083*** | 2.600*** | 2.821*** | 0.0340 |
|  | (0.847) | (0.766) | (0.693) | (0.649) | (0.453) |
| West Midlands | -0.537 | 0.563 | 0.685 | 1.256* | -0.417 |
|  | (1.031) | (0.967) | (0.731) | (0.717) | (0.471) |
| Yorkshire and the Humber | 5.149*** | 4.768*** | 2.545*** | 2.489*** | 0.520 |
|  | (1.115) | (1.033) | (0.832) | (0.792) | (0.556) |
| Constant | 55.82*** | 59.26*** | 35.72*** | 39.06*** | 31.10*** |
|  | (3.466) | (3.434) | (3.358) | (3.470) | (2.480) |
| Observations | 463 | 463 | 463 | 463 | 463 |
| R-squared | 0.684 | 0.717 | 0.815 | 0.826 | 0.918 |

*Note: Robust standard errors in parentheses; *** p<0.01, ** p<0.05, * p<0.1.*

## *Table S1.11. (cont.) Results for vaccination second doses with all controls and region fixed effects*

| **Model** | **6** | **7** | **8** | **9** | **10** |
| --- | --- | --- | --- | --- | --- |
| Conservative vote share | 0.0882*** | 0.0916*** | 0.102*** | 0.0856*** | 0.179*** |
|  | (0.0161) | (0.0229) | (0.0225) | (0.0203) | (0.0292) |
| Leave vote in EU Referendum | -0.198*** | -0.0147 | 0.0378 | -0.0706** | -0.146*** |
|  | (0.0205) | (0.0333) | (0.0329) | (0.0284) | (0.0529) |
| Median House Price | -0.0109*** | -0.00869*** | -0.0112*** | -0.00320 | -0.00333 |
| *(in 1000s)* | (0.00224) | (0.00226) | (0.00236) | (0.00243) | (0.00340) |
| Median wage | 0.0637* | -0.164*** | -0.116** | -0.122** | -0.112* |
| *(in 1000s)* | (0.0361) | (0.0552) | (0.0547) | (0.0534) | (0.0662) |
| Population density | -0.00102 | -4.19e-05 | -0.000982 | -0.00307*** | -0.00460*** |
|  | (0.000744) | (0.00118) | (0.00105) | (0.000982) | (0.00123) |
| Share of population White | 0.220*** | 0.291*** | 0.296*** | 0.294*** | 0.306*** |
|  | (0.0244) | (0.0279) | (0.0291) | (0.0260) | (0.0309) |
| Median age | 0.792*** |  |  |  |  |
|  | (0.0385) |  |  |  |  |
| IMD - Overall index rank |  | 0.0205*** |  |  |  |
|  |  | (0.00218) |  |  |  |
| IMD - Education, skills & training rank |  |  | 0.0212*** |  |  |
|  |  |  | (0.00198) |  |  |
| Share of deprived LSOAs |  |  |  | -0.194*** |  |
|  |  |  |  | (0.0179) |  |
| Share residents >16 years old with no qualification |  |  |  |  | -0.103 |
|  |  |  |  |  | (0.0895) |
| *Region fixed effects*  *(Ref: East of England)* |  |  |  |  |  |
| East Midlands | 0.299 | 1.237 | 0.482 | 1.905** | 1.670* |
|  | (0.692) | (0.847) | (0.890) | (0.876) | (0.955) |
| London | 0.747 | 3.145*** | 0.894 | 1.119 | 3.570*** |
|  | (0.808) | (1.062) | (1.023) | (1.034) | (1.132) |
| North East | 0.951 | 4.505*** | 1.786* | 4.876*** | 4.101*** |
|  | (0.693) | (0.968) | (1.011) | (0.908) | (1.068) |
| North West | -1.529*** | 2.089*** | -0.529 | 3.092*** | 1.552* |
|  | (0.563) | (0.725) | (0.805) | (0.772) | (0.834) |
| South East | 0.340 | 1.117** | 0.651 | 1.207** | 0.903 |
|  | (0.377) | (0.547) | (0.593) | (0.607) | (0.594) |
| South West | 0.473 | 3.092*** | 1.808*** | 3.093*** | 2.726*** |
|  | (0.474) | (0.620) | (0.638) | (0.654) | (0.670) |
| West Midlands | 0.0440 | 1.455** | -0.312 | 2.262*** | 1.394* |
|  | (0.477) | (0.636) | (0.665) | (0.680) | (0.741) |
| Yorkshire and the Humber | 0.293 | 2.339*** | 0.740 | 3.639*** | 2.469*** |
|  | (0.563) | (0.685) | (0.743) | (0.716) | (0.796) |
| Constant | 18.18*** | 32.11*** | 29.40*** | 40.53*** | 40.09*** |
|  | (2.463) | (3.002) | (3.297) | (2.881) | (3.483) |
| Observations | 463 | 463 | 463 | 463 | 463 |
| R-squared | 0.920 | 0.856 | 0.861 | 0.868 | 0.826 |

*Note: Robust standard errors in parentheses; *** p<0.01, ** p<0.05, * p<0.1.*

## *Table S1.12. Results for vaccination second doses regressed on votes share for non-Labour non-Conservative Parties*

| **Model** | **1** | **2** | **3** | **4** |
| --- | --- | --- | --- | --- |
| Vote share for neither Conservative nor Labour | 0.0155 | -0.0184 | -0.0253 | -0.00142 |
|  | (0.0209) | (0.0171) | (0.0171) | (0.0196) |
| Median House Price | 0.00934** | -0.00818*** | -0.0104*** | 0.00112 |
| *(in 1000s)* | (0.00363) | (0.00225) | (0.00247) | (0.00368) |
| Median wage | -0.0994 | -0.169*** | -0.112* | -0.133* |
| *(in 1000s)* | (0.0782) | (0.0591) | (0.0623) | (0.0752) |
| Population density | -0.00815*** | -0.00167 | -0.00421*** | -0.00737*** |
|  | (0.00125) | (0.00115) | (0.00107) | (0.00123) |
| Share of population White | 0.347*** | 0.323*** | 0.345*** | 0.352*** |
|  | (0.0302) | (0.0266) | (0.0291) | (0.0317) |
| IMD - Overall index rank |  | 0.0227*** |  |  |
|  |  | (0.00168) |  |  |
| IMD - Education, skills & training rank |  |  | 0.0201*** |  |
|  |  |  | (0.00148) |  |
| Share residents >16 years old with no qualification |  |  |  | -0.271*** |
|  |  |  |  | (0.0566) |
| *Region fixed effects (Ref: East of England)* |  |  |  |  |
| East Midlands | 2.222** | 1.193 | 0.481 | 1.943* |
|  | (1.009) | (0.845) | (0.918) | (1.014) |
| London | 3.112** | 3.568*** | 1.880* | 3.510*** |
|  | (1.262) | (1.072) | (1.052) | (1.206) |
| North East | 2.260** | 3.233*** | 0.0254 | 1.923* |
|  | (1.074) | (0.891) | (0.911) | (1.011) |
| North West | 0.368 | 0.925 | -2.216*** | -0.164 |
|  | (0.882) | (0.656) | (0.740) | (0.832) |
| South East | 0.817 | 0.996* | 0.461 | 0.480 |
|  | (0.644) | (0.563) | (0.621) | (0.647) |
| South West | 3.103*** | 2.886*** | 1.435** | 2.360*** |
|  | (0.674) | (0.605) | (0.652) | (0.718) |
| West Midlands | 2.033** | 1.791*** | 0.180 | 2.213*** |
|  | (0.796) | (0.635) | (0.681) | (0.761) |
| Yorkshire and the Humber | 1.326 | 1.571** | -0.251 | 1.188 |
|  | (0.930) | (0.669) | (0.723) | (0.834) |
| Constant | 31.82*** | 33.23*** | 33.51*** | 40.94*** |
|  | (3.765) | (3.039) | (3.298) | (3.875) |
| Observations | 463 | 463 | 463 | 463 |
| R-squared | 0.790 | 0.848 | 0.846 | 0.803 |

*Note: Robust standard errors in parentheses; *** p<0.01, ** p<0.05, * p<0.1.*

## *Table S1.13. Results for vaccination second doses and controlling for Labour vote share*

| **Model** | **1** | **2** | **3** | **4** |
| --- | --- | --- | --- | --- |
| Conservative vote share | 0.0624*** | 0.0606*** | 0.0862*** | 0.0767*** |
|  | (0.0231) | (0.0198) | (0.0193) | (0.0216) |
| Labour party vote share | -0.100*** | -0.0467** | -0.0553*** | -0.0903*** |
|  | (0.0220) | (0.0192) | (0.0185) | (0.0209) |
| Median House Price | 0.00442 | -0.00970*** | -0.0136*** | -0.00444 |
| *(in 1000s)* | (0.00369) | (0.00222) | (0.00225) | (0.00348) |
| Median wage | -0.103 | -0.164*** | -0.114** | -0.138** |
| *(in 1000s)* | (0.0666) | (0.0532) | (0.0527) | (0.0624) |
| Population density | -0.00374*** | 0.000524 | -0.000620 | -0.00281** |
|  | (0.00134) | (0.00115) | (0.00105) | (0.00126) |
| Share of population White | 0.279*** | 0.280*** | 0.285*** | 0.282*** |
|  | (0.0298) | (0.0282) | (0.0300) | (0.0315) |
| IMD - Overall index rank |  | 0.0202*** |  |  |
|  |  | (0.00162) |  |  |
| IMD - Education, skills |  |  | 0.0190*** |  |
| & training rank |  |  | (0.00141) |  |
| Share residents >16 years old |  |  |  | -0.282*** |
| with no qualification |  |  |  | (0.0509) |
| *Region fixed effects*  *(Ref: East of England)* |  |  |  |  |
| East Midlands | 2.160** | 1.270 | 0.539 | 1.873* |
|  | (0.996) | (0.840) | (0.879) | (0.976) |
| London | 2.466** | 3.079*** | 1.357 | 2.858** |
|  | (1.237) | (1.068) | (0.996) | (1.150) |
| North East | 4.391*** | 4.509*** | 2.005** | 4.071*** |
|  | (1.153) | (0.959) | (1.001) | (1.089) |
| North West | 2.612*** | 2.316*** | -0.144 | 2.095** |
|  | (0.922) | (0.728) | (0.810) | (0.875) |
| South East | 0.898 | 0.998* | 0.519 | 0.521 |
|  | (0.623) | (0.546) | (0.581) | (0.609) |
| South West | 3.166*** | 2.910*** | 1.553** | 2.355*** |
|  | (0.675) | (0.591) | (0.619) | (0.688) |
| West Midlands | 1.453* | 1.413** | -0.249 | 1.610** |
|  | (0.798) | (0.633) | (0.646) | (0.737) |
| Yorkshire and the Humber | 2.481*** | 2.283*** | 0.833 | 2.343*** |
|  | (0.925) | (0.685) | (0.727) | (0.818) |
| Constant | 38.45*** | 35.57*** | 36.08*** | 46.89*** |
|  | (3.910) | (3.283) | (3.421) | (3.844) |
| Observations | 463 | 463 | 463 | 463 |
| R-squared | 0.814 | 0.858 | 0.863 | 0.829 |

*Note: Robust standard errors in parentheses; *** p<0.01, ** p<0.05, * p<0.1.*

## *Table S1.14. Results for age-specific vaccination rates (first doses)*

| **Model** | **1** | **2** | **3** | **4** | **5** | **6** | **7** |
| --- | --- | --- | --- | --- | --- | --- | --- |
| Vaccination rate for age group: | <18 | 18-24 | 25-29 | 30-34 | 35-39 | 40-44 | 45-49 |
| Conservative vote share | 0.000161 | 0.00173*** | 0.00196*** | 0.00160*** | 0.00139*** | 0.00134*** | 0.00124*** |
|  | (0.000117) | (0.000290) | (0.000346) | (0.000317) | (0.000292) | (0.000257) | (0.000211) |
| Median House Price | 2.13e-05 | -6.09e-05 | 4.82e-05 | -1.06e-05 | -2.81e-05 | -6.18e-05 | -9.06e-05** |
| *(in 1000s)* | (1.57e-05) | (4.45e-05) | (6.46e-05) | (4.81e-05) | (4.46e-05) | (4.33e-05) | (3.99e-05) |
| Median wage | 2.02e-05 | -0.000529 | -0.000458 | -0.000810 | -0.00132 | -0.000948 | -0.000223 |
| *(in 1000s)* | (0.000260) | (0.000928) | (0.00102) | (0.000881) | (0.000885) | (0.000867) | (0.000761) |
| Population density | -3.13e-05*** | -4.36e-05*** | -2.67e-05 | -5.04e-05*** | -8.16e-05*** | -7.97e-05*** | -5.95e-05*** |
|  | (5.83e-06) | (1.56e-05) | (2.00e-05) | (1.65e-05) | (1.67e-05) | (1.77e-05) | (1.59e-05) |
| Share of population White | 0.000446*** | 0.00268*** | 0.00260*** | 0.00243*** | 0.00213*** | 0.00186*** | 0.00170*** |
|  | (0.000107) | (0.000424) | (0.000446) | (0.000402) | (0.000381) | (0.000335) | (0.000279) |
| Share residents >16 years old with no qualification | -0.00203*** | -0.00575*** | -0.00283*** | -0.00367*** | -0.00283*** | -0.00208*** | -0.00167*** |
|  | (0.000278) | (0.000729) | (0.00102) | (0.000816) | (0.000739) | (0.000657) | (0.000555) |
| *Region fixed effects*  *(Ref: East of England)* |  |  |  |  |  |  |  |
| East Midlands | 0.00952** | 0.0261** | 0.0126 | 0.0109 | 0.00679 | 0.00332 | 0.00208 |
|  | (0.00479) | (0.0130) | (0.0158) | (0.0147) | (0.0140) | (0.0120) | (0.00946) |
| London | -0.0114** | 0.000330 | 0.0288 | 0.0250 | 0.0268* | 0.0229* | 0.0155 |
|  | (0.00515) | (0.0156) | (0.0196) | (0.0163) | (0.0147) | (0.0130) | (0.0109) |
| North East | -0.00270 | 0.0427*** | 0.0496** | 0.0526*** | 0.0562*** | 0.0424*** | 0.0276*** |
|  | (0.00513) | (0.0152) | (0.0203) | (0.0173) | (0.0145) | (0.0125) | (0.0102) |
| North West | 0.00258 | 0.0114 | 0.0257* | 0.0252** | 0.0236** | 0.0148 | 0.00626 |
|  | (0.00519) | (0.0121) | (0.0138) | (0.0121) | (0.0115) | (0.0103) | (0.00849) |
| South East | -0.00541 | 0.00440 | 0.00971 | 0.0152 | 0.0195** | 0.0189** | 0.0125** |
|  | (0.00380) | (0.00921) | (0.0103) | (0.00937) | (0.00882) | (0.00750) | (0.00582) |
| South West | -0.0232*** | 0.0315*** | 0.0353*** | 0.0413*** | 0.0349*** | 0.0262*** | 0.0179*** |
|  | (0.00377) | (0.00962) | (0.0114) | (0.0103) | (0.00970) | (0.00840) | (0.00674) |
| West Midlands | -0.000511 | 0.0108 | 0.0120 | 0.0213** | 0.0169 | 0.00917 | 0.00462 |
|  | (0.00437) | (0.0103) | (0.0117) | (0.0104) | (0.0104) | (0.00927) | (0.00742) |
| Yorkshire and the Humber | -0.00331 | 0.0206* | 0.0292** | 0.0383*** | 0.0307*** | 0.0195* | 0.0146* |
|  | (0.00444) | (0.0112) | (0.0137) | (0.0114) | (0.0110) | (0.0100) | (0.00818) |
| Constant | 0.0950*** | 0.540*** | 0.405*** | 0.507*** | 0.591*** | 0.653*** | 0.697*** |
|  | (0.0145) | (0.0489) | (0.0587) | (0.0490) | (0.0477) | (0.0454) | (0.0399) |
| Observations | 463 | 463 | 463 | 463 | 463 | 463 | 463 |
| R-squared | 0.538 | 0.752 | 0.580 | 0.686 | 0.730 | 0.750 | 0.777 |

*Note: Robust standard errors in parentheses; *** p<0.01, ** p<0.05, * p<0.1.*

## *Table S1.14. (cont.) Results for age-specific vaccination rates (first doses)*

| **Model** | **8** | **9** | **10** | **11** | **12** | **13** | **14** |
| --- | --- | --- | --- | --- | --- | --- | --- |
| Vaccination rate for age group: | 50-54 | 55-59 | 60-64 | 65-69 | 70-74 | 75-79 | 80-84 |
| Conservative vote share | 0.000985*** | 0.000787*** | 0.000523*** | 0.000416*** | 0.000263*** | 0.000183*** | 0.000204*** |
|  | (0.000166) | (0.000136) | (0.000117) | (9.87e-05) | (8.20e-05) | (6.56e-05) | (7.45e-05) |
| Median House Price | -0.000102*** | -0.000101*** | -9.61e-05*** | -8.52e-05*** | -8.70e-05*** | -7.01e-05*** | -7.13e-05*** |
| *(in 1000s)* | (3.29e-05) | (2.80e-05) | (2.67e-05) | (2.50e-05) | (2.20e-05) | (1.66e-05) | (1.73e-05) |
| Median wage | -0.000128 | -0.000177 | -0.000204 | -0.000352 | -0.000220 | -6.05e-05 | 5.64e-05 |
| *(in 1000s)* | (0.000609) | (0.000523) | (0.000434) | (0.000376) | (0.000318) | (0.000290) | (0.000310) |
| Population density | -4.30e-05*** | -3.76e-05*** | -4.19e-05*** | -3.69e-05*** | -3.31e-05*** | -3.50e-05*** | -3.15e-05*** |
|  | (1.30e-05) | (1.13e-05) | (1.04e-05) | (8.64e-06) | (7.55e-06) | (6.38e-06) | (6.54e-06) |
| Share of population White | 0.00174*** | 0.00170*** | 0.00156*** | 0.00133*** | 0.00109*** | 0.000967*** | 0.00128*** |
|  | (0.000245) | (0.000228) | (0.000206) | (0.000175) | (0.000148) | (0.000126) | (0.000146) |
| Share residents >16 years old with no qualification | -0.00137*** | -0.000944*** | -0.000750** | -0.000672** | -0.000732*** | -0.000457** | -0.000604** |
|  | (0.000436) | (0.000363) | (0.000336) | (0.000300) | (0.000266) | (0.000204) | (0.000237) |
| *Region fixed effects*  *(Ref: East of England)* |  |  |  |  |  |  |  |
| East Midlands | -0.00159 | -0.00163 | -0.000165 | 0.00338 | 0.00152 | 0.000711 | -0.00182 |
|  | (0.00741) | (0.00598) | (0.00541) | (0.00431) | (0.00326) | (0.00255) | (0.00295) |
| London | 0.00791 | 0.00893 | 0.00980 | 0.00154 | -0.00752 | -0.0137*** | -0.0138** |
|  | (0.00887) | (0.00771) | (0.00703) | (0.00602) | (0.00524) | (0.00465) | (0.00541) |
| North East | 0.0184** | 0.0179*** | 0.0166*** | 0.0163*** | 0.00714* | 0.00582* | -8.46e-05 |
|  | (0.00779) | (0.00619) | (0.00567) | (0.00509) | (0.00422) | (0.00325) | (0.00354) |
| North West | 0.000194 | -0.000634 | -0.000207 | 0.00295 | -0.00259 | -0.00187 | -0.00540* |
|  | (0.00680) | (0.00573) | (0.00527) | (0.00465) | (0.00371) | (0.00284) | (0.00314) |
| South East | 0.00767* | 0.00763** | 0.00561* | 0.00618** | 0.00332 | 0.00177 | 0.000936 |
|  | (0.00445) | (0.00367) | (0.00325) | (0.00285) | (0.00202) | (0.00171) | (0.00176) |
| South West | 0.00942* | 0.00634 | 0.00448 | 0.00337 | -0.00106 | -0.000703 | -0.00209 |
|  | (0.00535) | (0.00453) | (0.00405) | (0.00347) | (0.00269) | (0.00216) | (0.00236) |
| West Midlands | 0.000902 | -0.00108 | 0.00160 | 0.00535 | 0.00238 | 0.00274 | -0.00444 |
|  | (0.00567) | (0.00504) | (0.00457) | (0.00395) | (0.00308) | (0.00258) | (0.00275) |
| Yorkshire and the Humber | 0.00742 | 0.00827 | 0.00773 | 0.00911** | 0.00345 | 0.00311 | -0.000972 |
|  | (0.00631) | (0.00527) | (0.00493) | (0.00429) | (0.00339) | (0.00269) | (0.00287) |
| Constant | 0.741*** | 0.765*** | 0.801*** | 0.838*** | 0.888*** | 0.899*** | 0.872*** |
|  | (0.0336) | (0.0304) | (0.0276) | (0.0249) | (0.0215) | (0.0182) | (0.0200) |
| Observations | 463 | 463 | 463 | 463 | 463 | 463 | 463 |
| R-squared | 0.820 | 0.846 | 0.856 | 0.876 | 0.887 | 0.900 | 0.891 |

*Note: Robust standard errors in parentheses; *** p<0.01, ** p<0.05, * p<0.1.*

## *Table S1.15. Results for age-specific vaccination rates (second doses)*

| **Model** | **1** | **2** | **3** | **4** | **5** | **6** | **7** |
| --- | --- | --- | --- | --- | --- | --- | --- |
| Dependent variable | <18 | 18-24 | 25-29 | 30-34 | 35-39 | 40-44 | 45-49 |
| Conservative vote share | 0.00162*** | 0.00173*** | 0.00150*** | 0.00141*** | 0.00145*** | 0.00138*** | 0.00112*** |
|  | (0.000294) | (0.000345) | (0.000318) | (0.000301) | (0.000271) | (0.000225) | (0.000180) |
| Median House Price | -5.32e-05 | 5.99e-05 | -4.50e-06 | -2.12e-05 | -5.55e-05 | -8.65e-05* | -0.000106*** |
| *(in 1000s)* | (5.01e-05) | (7.11e-05) | (5.45e-05) | (5.10e-05) | (5.03e-05) | (4.51e-05) | (3.69e-05) |
| Median wage | -0.000356 | -0.000252 | -0.000736 | -0.00126 | -0.000981 | -0.000238 | -8.12e-05 |
| *(in 1000s)* | (0.000911) | (0.00102) | (0.000915) | (0.000945) | (0.000934) | (0.000825) | (0.000660) |
| Population density | -4.57e-05*** | -2.53e-05 | -4.73e-05*** | -8.40e-05*** | -8.34e-05*** | -6.62e-05*** | -4.97e-05*** |
|  | (1.54e-05) | (2.02e-05) | (1.71e-05) | (1.75e-05) | (1.87e-05) | (1.67e-05) | (1.38e-05) |
| Share of population White | 0.00301*** | 0.00299*** | 0.00277*** | 0.00245*** | 0.00220*** | 0.00207*** | 0.00206*** |
|  | (0.000440) | (0.000461) | (0.000417) | (0.000406) | (0.000370) | (0.000313) | (0.000270) |
| Share residents >16 years old with no qualification | -0.00681*** | -0.00417*** | -0.00491*** | -0.00405*** | -0.00326*** | -0.00258*** | -0.00195*** |
|  | (0.000754) | (0.00103) | (0.000847) | (0.000787) | (0.000725) | (0.000616) | (0.000493) |
| *Region fixed effects*  *(Ref: East of England)* |  |  |  |  |  |  |  |
| East Midlands | 0.0319** | 0.0197 | 0.0150 | 0.0122 | 0.00801 | 0.00677 | 0.000716 |
|  | (0.0138) | (0.0163) | (0.0150) | (0.0145) | (0.0129) | (0.0103) | (0.00819) |
| London | -0.000640 | 0.0290 | 0.0266 | 0.0297* | 0.0258* | 0.0203* | 0.00978 |
|  | (0.0158) | (0.0197) | (0.0169) | (0.0156) | (0.0142) | (0.0119) | (0.00965) |
| North East | 0.0325** | 0.0395* | 0.0423** | 0.0496*** | 0.0384*** | 0.0280** | 0.0201** |
|  | (0.0158) | (0.0204) | (0.0177) | (0.0155) | (0.0139) | (0.0114) | (0.00866) |
| North West | 0.00733 | 0.0181 | 0.0154 | 0.0168 | 0.00936 | 0.00297 | -0.00295 |
|  | (0.0129) | (0.0145) | (0.0128) | (0.0122) | (0.0112) | (0.00930) | (0.00754) |
| South East | -0.000745 | 0.00544 | 0.0108 | 0.0169* | 0.0176** | 0.0125** | 0.00785 |
|  | (0.00901) | (0.0101) | (0.00944) | (0.00904) | (0.00787) | (0.00618) | (0.00486) |
| South West | 0.0243** | 0.0282** | 0.0351*** | 0.0324*** | 0.0252*** | 0.0179** | 0.00947 |
|  | (0.0100) | (0.0117) | (0.0106) | (0.0101) | (0.00892) | (0.00725) | (0.00582) |
| West Midlands | 0.0136 | 0.0136 | 0.0211** | 0.0174 | 0.00907 | 0.00723 | 0.00117 |
|  | (0.0104) | (0.0117) | (0.0106) | (0.0108) | (0.00980) | (0.00795) | (0.00624) |
| Yorkshire and the Humber | 0.0167 | 0.0267* | 0.0342*** | 0.0304*** | 0.0214** | 0.0181** | 0.00940 |
|  | (0.0116) | (0.0140) | (0.0120) | (0.0117) | (0.0109) | (0.00887) | (0.00702) |
| Constant | 0.426*** | 0.324*** | 0.441*** | 0.534*** | 0.603*** | 0.645*** | 0.695*** |
|  | (0.0498) | (0.0597) | (0.0512) | (0.0512) | (0.0499) | (0.0439) | (0.0369) |
| Observations | 463 | 463 | 463 | 463 | 463 | 463 | 463 |
| R-squared | 0.766 | 0.609 | 0.693 | 0.738 | 0.760 | 0.791 | 0.832 |

*Note: Robust standard errors in parentheses; *** p<0.01, ** p<0.05, * p<0.1.*

## *Table S1.15. (cont.) Results for age-specific vaccination rates (second doses)*

| **Model** | **8** | **9** | **10** | **11** | **12** | **13** |
| --- | --- | --- | --- | --- | --- | --- |
| Dependent variable | 50-54 | 55-59 | 60-64 | 65-69 | 70-74 | 75-79 |
| Conservative vote share | 0.000869*** | 0.000594*** | 0.000474*** | 0.000307*** | 0.000253*** | 0.000310*** |
|  | (0.000149) | (0.000126) | (0.000103) | (8.41e-05) | (7.65e-05) | (8.45e-05) |
| Median House Price | -0.000114*** | -0.000107*** | -9.55e-05*** | -9.76e-05*** | -7.79e-05*** | -7.77e-05*** |
| *(in 1000s)* | (3.09e-05) | (2.93e-05) | (2.70e-05) | (2.42e-05) | (2.00e-05) | (2.15e-05) |
| Median wage | -3.28e-05 | -4.16e-05 | -0.000356 | -0.000282 | -0.000102 | 0.000106 |
| *(in 1000s)* | (0.000567) | (0.000463) | (0.000407) | (0.000339) | (0.000311) | (0.000348) |
| Population density | -4.64e-05*** | -5.14e-05*** | -4.28e-05*** | -3.77e-05*** | -4.11e-05*** | -3.62e-05*** |
|  | (1.21e-05) | (1.08e-05) | (8.83e-06) | (7.49e-06) | (6.48e-06) | (6.84e-06) |
| Share of population White | 0.00193*** | 0.00173*** | 0.00152*** | 0.00125*** | 0.00112*** | 0.00146*** |
|  | (0.000244) | (0.000224) | (0.000187) | (0.000156) | (0.000148) | (0.000160) |
| Share residents >16 years old with no qualification | -0.00131*** | -0.00103*** | -0.000917*** | -0.000931*** | -0.000638*** | -0.000847*** |
|  | (0.000409) | (0.000373) | (0.000328) | (0.000292) | (0.000245) | (0.000282) |
| *Region fixed effects*  *(Ref: East of England)* |  |  |  |  |  |  |
| East Midlands | -0.00365 | 0.000246 | 0.00376 | 0.00162 | 0.000281 | -0.00387 |
|  | (0.00647) | (0.00592) | (0.00465) | (0.00355) | (0.00300) | (0.00345) |
| London | 0.00790 | 0.0130* | 0.00179 | -0.00653 | -0.0117** | -0.0145** |
|  | (0.00824) | (0.00750) | (0.00630) | (0.00557) | (0.00542) | (0.00612) |
| North East | 0.0187*** | 0.0190*** | 0.0161*** | 0.00753 | 0.00692* | 0.000717 |
|  | (0.00686) | (0.00622) | (0.00551) | (0.00460) | (0.00390) | (0.00451) |
| North West | -0.00654 | -0.000819 | 0.000950 | -0.00410 | -0.00354 | -0.00826** |
|  | (0.00642) | (0.00577) | (0.00503) | (0.00407) | (0.00335) | (0.00381) |
| South East | 0.00660 | 0.00665* | 0.00619** | 0.00334 | 0.00184 | -0.000236 |
|  | (0.00404) | (0.00344) | (0.00309) | (0.00221) | (0.00200) | (0.00212) |
| South West | 0.00417 | 0.00255 | 0.00276 | -0.00132 | -0.000590 | -0.00165 |
|  | (0.00501) | (0.00435) | (0.00378) | (0.00301) | (0.00252) | (0.00283) |
| West Midlands | -0.00464 | 0.00428 | 0.00541 | 0.00251 | 0.00301 | -0.00319 |
|  | (0.00555) | (0.00488) | (0.00425) | (0.00335) | (0.00304) | (0.00311) |
| Yorkshire and the Humber | 0.00907 | 0.0104* | 0.00975** | 0.00491 | 0.00440 | 0.000442 |
|  | (0.00577) | (0.00529) | (0.00461) | (0.00374) | (0.00326) | (0.00351) |
| Constant | 0.730*** | 0.768*** | 0.816*** | 0.871*** | 0.881*** | 0.847*** |
|  | (0.0330) | (0.0296) | (0.0270) | (0.0230) | (0.0205) | (0.0227) |
| Observations | 463 | 463 | 463 | 463 | 463 | 463 |
| R-squared | 0.854 | 0.864 | 0.888 | 0.897 | 0.898 | 0.888 |

*Note: Robust standard errors in parentheses; *** p<0.01, ** p<0.05, * p<0.1.*

## *Table S1.16. Results for Conservative vote share and vaccination rate (first dose) - sample restrictions*

| **Model** | **1** | **2** | **3** | **4** | **5** | **6** | **7** | **8** | **9** |
| --- | --- | --- | --- | --- | --- | --- | --- | --- | --- |
| Sample restriction variable | House Prices | | | Median wages | | | Share deprived Local Authorities | | |
| Variable level restriction applied (tertile) | Low | Middle | High | Low | Middle | High | Low | Middle | High |
| Share conservative vote | 0.267*** | 0.271*** | 0.388*** | 0.269*** | 0.272*** | 0.382*** | 0.402*** | 0.350*** | 0.242*** |
|  | (0.0305) | (0.0418) | (0.0211) | (0.0371) | (0.0267) | (0.0261) | (0.0336) | (0.0248) | (0.0294) |
| Constant | 54.60*** | 55.94*** | 47.05*** | 56.08*** | 55.06*** | 46.91*** | 48.35*** | 50.26*** | 54.31*** |
|  | (1.476) | (2.396) | (1.052) | (2.028) | (1.405) | (1.266) | (1.898) | (1.368) | (1.368) |
| Observations | 181 | 174 | 177 | 155 | 154 | 154 | 189 | 167 | 176 |
| R-squared | 0.309 | 0.293 | 0.625 | 0.346 | 0.389 | 0.589 | 0.504 | 0.509 | 0.273 |

*Note: Robust standard errors in parentheses; *** p<0.01, ** p<0.05, * p<0.1.*

## *Table S1.16. (cont.) Conservative vote share and vaccination rate (first dose) - sample restrictions*

| **Model** | **10** | **11** | **12** | **13** | **14** | **15** | **16** | **17** | **18** |
| --- | --- | --- | --- | --- | --- | --- | --- | --- | --- |
| Sample restriction variable | Share no education | | | Share White Population | | | IMD Overall rank | | |
| Variable level restriction applied (tertile) | Low | Middle | High | Low | Middle | High | Low | Middle | High |
| Share conservative vote | 0.393*** | 0.383*** | 0.243*** | 0.284*** | 0.121*** | 0.108*** | 0.280*** | 0.301*** | 0.255*** |
|  | (0.0215) | (0.0295) | (0.0297) | (0.0239) | (0.0214) | (0.0219) | (0.0264) | (0.0285) | (0.0446) |
| Constant | 48.87*** | 48.98*** | 54.82*** | 48.37*** | 62.68*** | 67.57*** | 51.89*** | 52.35*** | 57.50*** |
|  | (1.123) | (1.647) | (1.583) | (0.923) | (1.117) | (1.182) | (1.146) | (1.584) | (2.575) |
| Observations | 188 | 173 | 171 | 178 | 177 | 177 | 178 | 177 | 177 |
| R-squared | 0.590 | 0.527 | 0.294 | 0.465 | 0.180 | 0.197 | 0.344 | 0.356 | 0.255 |

*Note: Robust standard errors in parentheses; *** p<0.01, ** p<0.05, * p<0.1.*

## *Table S1.16. (cont.) Results for Conservative vote share and vaccination rate (first dose) - sample restrictions*

| **Model** | **19** | **20** | **21** | **25** | **26** | **27** | **28** | **29** | **30** |
| --- | --- | --- | --- | --- | --- | --- | --- | --- | --- |
| Sample restriction variable | IMD Education rank | | | Share over 60 years old | | | Median age population | | |
| Variable level restriction applied (tertile) | Low | Middle | High | Low | Middle | High | Low | Middle | High |
| Share conservative vote | 0.251*** | 0.349*** | 0.397*** | 0.249*** | 0.0558*** | 0.0134 | 0.227*** | 0.0356* | 0.00151 |
|  | (0.0263) | (0.0307) | (0.0256) | (0.0246) | (0.0189) | (0.0220) | (0.0240) | (0.0194) | (0.0236) |
| Constant | 53.21*** | 50.98*** | 49.45*** | 49.94*** | 65.51*** | 73.26*** | 50.91*** | 66.59*** | 74.02*** |
|  | (1.349) | (1.727) | (1.407) | (0.939) | (0.988) | (1.199) | (0.940) | (1.051) | (1.297) |
| Observations | 178 | 177 | 177 | 178 | 177 | 177 | 182 | 176 | 174 |
| R-squared | 0.337 | 0.504 | 0.555 | 0.350 | 0.052 | 0.003 | 0.304 | 0.017 | 0.000 |

*Note: Robust standard errors in parentheses; *** p<0.01, ** p<0.05, * p<0.1.*

## *Table S1.17. Results for Conservative vote share and vaccination rate (second dose) - sample restrictions*

| **Model** | **1** | **2** | **3** | **4** | **5** | **6** | **7** | **8** | **9** |
| --- | --- | --- | --- | --- | --- | --- | --- | --- | --- |
| Sample restriction variable | House Prices | | | Median wages | | | Share deprived Local Authorities | | |
| Variable level restriction applied (tertile) | Low | Middle | High | Low | Middle | High | Low | Middle | High |
| Share conservative vote | 0.290*** | 0.291*** | 0.405*** | 0.295*** | 0.291*** | 0.402*** | 0.420*** | 0.367*** | 0.261*** |
|  | (0.0325) | (0.0448) | (0.0217) | (0.0393) | (0.0281) | (0.0272) | (0.0351) | (0.0254) | (0.0313) |
| Constant | 48.34*** | 50.04*** | 41.25*** | 49.85*** | 49.11*** | 40.84*** | 42.63*** | 44.50*** | 48.15*** |
|  | (1.579) | (2.571) | (1.075) | (2.156) | (1.476) | (1.318) | (1.984) | (1.405) | (1.455) |
| Observations | 181 | 174 | 177 | 155 | 154 | 154 | 189 | 167 | 176 |
| R-squared | 0.318 | 0.294 | 0.631 | 0.361 | 0.400 | 0.590 | 0.502 | 0.514 | 0.281 |

*Note: Robust standard errors in parentheses; *** p<0.01, ** p<0.05, * p<0.1.*

## *Table S1.17. (cont.) Results for Conservative vote share and vaccination rate (second dose) - sample restrictions*

| **Model** | **10** | **11** | **12** | **13** | **14** | **15** | **16** | **17** | **18** |
| --- | --- | --- | --- | --- | --- | --- | --- | --- | --- |
| Sample restriction variable | Share no education | | | Share White Population | | | IMD Overall rank | | |
| Variable level restriction applied (tertile) | Low | Middle | High | Low | Middle | High | Low | Middle | High |
| Share conservative vote | 0.411*** | 0.410*** | 0.262*** | 0.300*** | 0.137*** | 0.118*** | 0.296*** | 0.317*** | 0.270*** |
|  | (0.0220) | (0.0308) | (0.0316) | (0.0251) | (0.0225) | (0.0226) | (0.0277) | (0.0294) | (0.0457) |
| Constant | 43.08*** | 42.65*** | 48.68*** | 42.30*** | 56.85*** | 62.36*** | 45.86*** | 46.62*** | 51.89*** |
|  | (1.140) | (1.719) | (1.682) | (0.975) | (1.175) | (1.217) | (1.204) | (1.629) | (2.633) |
| Observations | 188 | 173 | 171 | 178 | 177 | 177 | 178 | 177 | 177 |
| R-squared | 0.595 | 0.537 | 0.303 | 0.473 | 0.200 | 0.206 | 0.347 | 0.361 | 0.259 |

*Note: Robust standard errors in parentheses; *** p<0.01, ** p<0.05, * p<0.1.*

## *Table S1.17. (cont.) Results for Conservative vote share and vaccination rate (second dose) - sample restrictions*

| **Model** | **19** | **20** | **21** | **25** | **26** | **27** | **28** | **29** | **30** |
| --- | --- | --- | --- | --- | --- | --- | --- | --- | --- |
| Sample restriction variable | IMD Education rank | | | Share over 60 years old | | | Median age population | | |
| Variable level restriction applied (tertile) | Low | Middle | High | Low | Middle | High | Low | Middle | High |
| Share conservative vote | 0.270*** | 0.374*** | 0.410*** | 0.263*** | 0.0639*** | 0.0175 | 0.240*** | 0.0435** | 0.00231 |
|  | (0.0277) | (0.0315) | (0.0263) | (0.0256) | (0.0191) | (0.0234) | (0.0251) | (0.0199) | (0.0247) |
| Constant | 46.97*** | 44.75*** | 43.99*** | 43.93*** | 60.03*** | 68.47*** | 44.94*** | 61.15*** | 69.41*** |
|  | (1.427) | (1.774) | (1.440) | (0.982) | (1.001) | (1.283) | (0.986) | (1.076) | (1.365) |
| Observations | 178 | 177 | 177 | 178 | 177 | 177 | 182 | 176 | 174 |
| R-squared | 0.348 | 0.516 | 0.555 | 0.360 | 0.063 | 0.004 | 0.312 | 0.024 | 0.000 |

*Note: Robust standard errors in parentheses; *** p<0.01, ** p<0.05, * p<0.1.*

# **S2. Individual level analyses**

## *Table S2.1. Summary Statistics YouGov survey*

| **Variables** | **(1)** | **(2)** | **(3)** |
| --- | --- | --- | --- |
|  | **N** | **Mean** | **SD** |
| *Dependent variable: Vaccinated* |  |  |  |
| Binary variable: 1 at least one dose received; 0 otherwise | 8,485 | 0.924 | 0.265 |
| Binary variable: 1 both injections required to be fully vaccinated against COVID–19; 0 otherwise | 8,485 | 0.849735 | 0.357352 |
| *Main independent variable: Vote in last general election (2019)* |  |  |  |
| Conservative party | 6,208 | 0.476643 | 0.499494 |
| *Controls* |  |  |  |
| Age | 8,485 | 51.23359 | 16.68259 |
| Gender (Male gender coded 1, 0 otherwise) | 8,485 | 0.42911 | 0.494978 |
| Number children (NB: 3 and above coded 3) | 8,143 | 0.379713 | 0.777977 |
| C2 D E class (coded 1, 0 otherwise) | 8,485 | 0.40495 | 0.490911 |
| Brexiter (coded 1 if voted to leave European Union in Referendum) | 8,311 | 0.413187 | 0.492436 |

## *Table S2.2. Past vote variable*

| **Which political party did you voted for in the last general election (2019)** | **Frequency** | **Percent** |
| --- | --- | --- |
| Brexit Party | 135 | 2.17% |
| Conservative | 2,959 | 48.72% |
| Green | 215 | 2.31% |
| Labour | 2,103 | 13.85% |
| Liberal Democrat | 796 | 3.55% |
| TOTAL | 6208 | 100% |

*Note: consistent with outcome of the election, the plurality although not majority of respondents voted for conservatives. This was followed by Labour voters, and quite far behind the LibDem, the other parties all received less than 5% and the conservative-labour voters together account for over 70% of the votes.*

## *Table S2.3. Vaccination status*

| **What is your vaccination status?** | **Frequency** | **Percent** | **Cumulative Percentage** |
| --- | --- | --- | --- |
| I have received all the injections required to be fully vaccinated against COVID–19 | 7,210 | 84.97 | 84.97 |
| I have started the vaccination process, but need another shot | 631 | 7.44 | 92.41 |
| I plan to get vaccinated | 104 | 1.23 | 93.64 |
| I will not get vaccinated | 323 | 3.81 | 97.44 |
| I’m not sure about getting vaccinated | 217 | 2.56 | 100 |
| **Total** | 8,485 | 100 |  |

*Note: YouGov fielded a specific question about whether respondents had already vaccinated, and if so how many doses did they receive, and if not, whether they planned to did not plan to get vaccinated. It is noteworthy that at the time of the survey, nearly 85% had received both recommended doses at the time. It is important to recall the age restrictions on YouGov samples, i.e. people are above 18 and hence 85% is NOT the percentage of population that has received both doses. While there are fewer respondents do not want to get vaccinated or hesitate (5.3%), this represents a sufficiently large number for most purposes (540 respondents out of 8485). Finally, note that the entirety of our sample responded to this question, whereas as is common in survey of political preferences, fewer responded to question about past vote (around 6.8k).*

## *Table S2.4. Regions*

| **Regional distribution of survey responses** | **Frequency** | **Percent** | **Cumulative Percentage** |
| --- | --- | --- | --- |
| East Midlands | 687 | 8.1 | 8.1 |
| East of England | 850 | 10.02 | 18.11 |
| London | 885 | 10.43 | 28.54 |
| North East | 395 | 4.66 | 33.2 |
| North West | 890 | 10.49 | 43.69 |
| Scotland | 761 | 8.97 | 52.66 |
| South East | 1,227 | 14.46 | 67.12 |
| South West | 863 | 10.17 | 77.29 |
| Wales | 397 | 4.68 | 81.97 |
| West Midlands | 749 | 8.83 | 90.8 |
| Yorkshire and the Humber | 781 | 9.2 | 100 |
| Total | 8,485 | 100 |  |

*Note: There are substantial respondents in regions of the UK with the southeast being slightly over-represented, but we checked whether inclusion of region fixed effects capturing unobservable cross-sectional heterogeneity changed effects and it does not.*

## *Table S2.5. Results for vaccination status (at least one dose) and past conservative vote*

| **Model** | **1** | **2** | **3** |
| --- | --- | --- | --- |
| **Dependent variable** | **Vaccinated at least once** | | |
| **Estimation method** | **OLS** | **OLS** | **Logistic** |
| Voted Conservative at last General Election | 0.0115* | 0.0126* | 0.250* |
| *(binary variable)* | (0.00653) | (0.00655) | (0.144) |
|  |  |  |  |
| Age of respondent | 0.00174*** | 0.00174*** | 0.0429*** |
|  | (0.000209) | (0.000210) | (0.00465) |
| Male respondent | -0.00284 | -0.00299 | -0.0885 |
| *(binary variable)* | (0.00546) | (0.00546) | (0.125) |
| Number of Children | -0.0114** | -0.0114** | -0.181** |
|  | (0.00525) | (0.00527) | (0.0747) |
| C2DE class | -0.0323*** | -0.0327*** | -0.711*** |
| *(binary variable)* | (0.00651) | (0.00658) | (0.135) |
| Voted Leave | -0.0466*** | -0.0473*** | -1.112*** |
| *(binary variable)* | (0.00698) | (0.00699) | (0.154) |
|  |  |  |  |
| *Regional dummy variables*  *(Ref: East Midlands)* |  |  |  |
| East of England |  | 0.00106 | 0.0324 |
| *(binary variable)* |  | (0.0126) | (0.289) |
| London |  | -0.00153 | -0.0411 |
| *(binary variable)* |  | (0.0127) | (0.279) |
| North East |  | 0.0173 | 0.492 |
| *(binary variable)* |  | (0.0138) | (0.403) |
| North West |  | 0.00161 | 0.0430 |
| *(binary variable)* |  | (0.0123) | (0.279) |
| Scotland |  | -0.0112 | -0.222 |
| *(binary variable)* |  | (0.0155) | (0.331) |
| South East |  | -0.00843 | -0.179 |
| *(binary variable)* |  | (0.0118) | (0.257) |
| South West |  | 0.000644 | 0.0581 |
| *(binary variable)* |  | (0.0122) | (0.283) |
| Wales |  | -0.0122 | -0.198 |
| *(binary variable)* |  | (0.0177) | (0.345) |
| West Midlands |  | -0.00421 | -0.0794 |
| *(binary variable)* |  | (0.0133) | (0.285) |
| Yorkshire and the Humber |  | 0.00169 | 0.0707 |
| *(binary variable)* |  | (0.0130) | (0.295) |
| Constant | 0.891*** | 0.893*** | 1.776*** |
|  | (0.0120) | (0.0154) | (0.301) |
| Observations | 5,967 | 5,967 | 5,967 |
| R-squared | 0.030 | 0.030 |  |

*Note: Robust standard errors in parentheses; *** p<0.01, ** p<0.05, * p<0.1.*

## *Table S2.6. Results for regressions of conservative vote share on vaccination status with age specific sub-samples*

| **Sample restrictions:** | **Age < 25** | **25 < Age < 55** | **Age > 55** |
| --- | --- | --- | --- |
| **Model** | **1** | **2** | **3** |
| **Dependent variable** | **Vaccinated at least once** | | |
| Voted Conservative  at last General Election | -0.0692 | 0.0210*** | 0.0164** |
| *(binary variable)* | (0.0457) | (0.00652) | (0.00707) |
| Male respondent | -0.0157 | 0.00140 | -0.00603 |
| *(binary variable)* | (0.0341) | (0.00545) | (0.00597) |
| Number of Children | -0.192*** | -0.0224*** | -0.0344 |
|  | (0.0558) | (0.00505) | (0.0240) |
| C2DE class | -0.111** | -0.0291*** | -0.00861 |
| *(binary variable)* | (0.0481) | (0.00657) | (0.00633) |
| Voted Leave | 0.0960 | -0.0377*** | -0.0339*** |
| *(binary variable)* | (0.0671) | (0.00694) | (0.00664) |
| *Regional dummy variables (Ref: East Midlands)* | |  |  |
| East of England | 0.0208 | 0.000551 | -0.0248 |
| *(binary variable)* | (0.0777) | (0.0127) | (0.0152) |
| London | -0.0204 | -0.00696 | -0.00501 |
| *(binary variable)* | (0.0797) | (0.0128) | (0.0135) |
| North East | -0.0462 | 0.0207 | 0.0248** |
| *(binary variable)* | (0.107) | (0.0140) | (0.00971) |
| North West | -0.000101 | 0.00317 | 0.00607 |
| *(binary variable)* | (0.0895) | (0.0124) | (0.0117) |
| Scotland | 0.0173 | -0.00826 | -0.0115 |
| *(binary variable)* | (0.112) | (0.0157) | (0.0165) |
| South East | -0.0606 | -0.00686 | -0.00484 |
| *(binary variable)* | (0.0828) | (0.0120) | (0.0119) |
| South West | -0.0277 | 0.00284 | -0.00940 |
| *(binary variable)* | (0.0797) | (0.0124) | (0.0131) |
| Wales | -0.0223 | -0.0108 | -0.0376* |
| *(binary variable)* | (0.102) | (0.0177) | (0.0228) |
| West Midlands | -0.0102 | -0.00486 | -0.00498 |
| *(binary variable)* | (0.0803) | (0.0135) | (0.0137) |
| Yorkshire and the Humber | 0.0813 | 0.00108 | -0.00178 |
| *(binary variable)* | (0.0801) | (0.0132) | (0.0133) |
| Constant | 0.969*** | 0.978*** | 0.997*** |
|  | (0.0653) | (0.0102) | (0.0101) |
| Observations | 312 | 5,967 | 2,926 |
| R-squared | 0.150 | 0.017 | 0.018 |

*Note: Robust standard errors in parentheses; *** p<0.01, ** p<0.05, * p<0.1.*

## *Table S2.7. Results for vaccination status (at least one dose) and conservative vote - region sub-samples*

| **Sample restrictions** | **East of England** | **London** | **North East** | **North West** | **Scotland** |
| --- | --- | --- | --- | --- | --- |
| **Model** | **1** | **2** | **3** | **4** | **5** |
| Voted Conservative | 0.0114* | 0.0115* | 0.0123* | 0.0117* | 0.0115* |
| *(binary variable)* | (0.00652) | (0.00654) | (0.00656) | (0.00650) | (0.00653) |
| Male respondent | -0.00282 | -0.00284 | -0.00298 | -0.00284 | -0.00280 |
| *(binary variable)* | (0.00546) | (0.00545) | (0.00546) | (0.00546) | (0.00546) |
| Number of Children | -0.0114** | -0.0114** | -0.0114** | -0.0114** | -0.0114** |
|  | (0.00524) | (0.00527) | (0.00525) | (0.00525) | (0.00525) |
| C2DE class | -0.0324*** | -0.0323*** | -0.0325*** | -0.0324*** | -0.0324*** |
| *(binary variable)* | (0.00652) | (0.00655) | (0.00651) | (0.00651) | (0.00652) |
| Voted Leave | -0.0466*** | -0.0466*** | -0.0472*** | -0.0467*** | -0.0468*** |
| *(binary variable)* | (0.00698) | (0.00698) | (0.00702) | (0.00697) | (0.00699) |
| Age respondent | 0.00174*** | 0.00174*** | 0.00173*** | 0.00174*** | 0.00174*** |
|  | (0.000209) | (0.000210) | (0.000209) | (0.000209) | (0.000209) |

*Note: Robust standard errors in parentheses; *** p<0.01, ** p<0.05, * p<0.1.*

## *Table S2.7. (cont.) Results for vaccination status (at least one dose) and conservative vote - region sub-samples*

| **Sample restrictions** | **South East** | **South West** | **Wales** | **West Midlands** | **Yorkshire and the Humber** |
| --- | --- | --- | --- | --- | --- |
| **Model** | **6** | **7** | **8** | **9** | **10** |
| Voted Conservative | 0.0119* | 0.0114* | 0.0112* | 0.0116* | 0.0115* |
| *(binary variable)* | (0.00655) | (0.00653) | (0.00652) | (0.00653) | (0.00653) |
| Male respondent | -0.00298 | -0.00282 | -0.00274 | -0.00285 | -0.00287 |
| *(binary variable)* | (0.00545) | (0.00546) | (0.00545) | (0.00545) | (0.00546) |
| Number of Children | -0.0114** | -0.0114** | -0.0114** | -0.0114** | -0.0114** |
|  | (0.00524) | (0.00525) | (0.00525) | (0.00525) | (0.00525) |
| C2DE class | -0.0325*** | -0.0324*** | -0.0322*** | -0.0324*** | -0.0324*** |
| *(binary variable)* | (0.00650) | (0.00651) | (0.00652) | (0.00652) | (0.00652) |
| Voted Leave | -0.0468*** | -0.0466*** | -0.0465*** | -0.0466*** | -0.0466*** |
| *(binary variable)* | (0.00699) | (0.00698) | (0.00697) | (0.00698) | (0.00698) |
| Age respondent | 0.00174*** | 0.00174*** | 0.00174*** | 0.00174*** | 0.00174*** |
|  | (0.000209) | (0.000209) | (0.000209) | (0.000209) | (0.000209) |

*Note: Robust standard errors in parentheses; *** p<0.01, ** p<0.05, * p<0.1.*

## *Table S2.8. Results for agreement that Government handled Covid-19 well regressed on Conservative vote*

| **Model** | **1** | **2** | **3** | **4** | **5** | **6** | **7** |
| --- | --- | --- | --- | --- | --- | --- | --- |
| Voted Conservative at last General Election | 0.502*** | 0.489*** | 0.489*** | 0.487*** | 0.485*** | 0.455*** | 0.456*** |
| *(binary variable)* | (0.0112) | (0.0118) | (0.0118) | (0.0120) | (0.0120) | (0.0146) | (0.0148) |
| Age of respondent |  | 0.00148*** | 0.00146*** | 0.00138*** | 0.00129*** | 0.00105*** | 0.00110*** |
|  |  | (0.000358) | (0.000360) | (0.000377) | (0.000378) | (0.000384) | (0.000385) |
| Male respondent |  |  | 0.00573 | 0.00851 | 0.00931 | 0.00919 | 0.00839 |
| *(binary variable)* |  |  | (0.0112) | (0.0114) | (0.0114) | (0.0114) | (0.0114) |
| Number of Children |  |  |  | -0.00100 | -0.000349 | -0.00429 | -0.00350 |
|  |  |  |  | (0.00836) | (0.00837) | (0.00838) | (0.00841) |
| C2DE class |  |  |  |  | 0.0295** | 0.0207* | 0.0207* |
| *(binary variable)* |  |  |  |  | (0.0120) | (0.0122) | (0.0122) |
| Voted Leave |  |  |  |  |  | 0.0621*** | 0.0634*** |
| *(binary variable)* |  |  |  |  |  | (0.0150) | (0.0151) |
| *Regional dummy variables (Ref: East Midlands)* |  |  |  |  |  |  |  |
| East of England |  |  |  |  |  |  | 0.0156 |
| *(binary variable)* |  |  |  |  |  |  | (0.0255) |
| London |  |  |  |  |  |  | 0.0411 |
| *(binary variable)* |  |  |  |  |  |  | (0.0256) |
| North East |  |  |  |  |  |  | 0.0187 |
| *(binary variable)* |  |  |  |  |  |  | (0.0319) |
| North West |  |  |  |  |  |  | 0.0123 |
| *(binary variable)* |  |  |  |  |  |  | (0.0248) |
| Scotland |  |  |  |  |  |  | 0.0395 |
| *(binary variable)* |  |  |  |  |  |  | (0.0326) |
| South East |  |  |  |  |  |  | 0.0244 |
| *(binary variable)* |  |  |  |  |  |  | (0.0235) |
| South West |  |  |  |  |  |  | 0.00531 |
| *(binary variable)* |  |  |  |  |  |  | (0.0253) |
| Wales |  |  |  |  |  |  | -0.00132 |
| *(binary variable)* |  |  |  |  |  |  | (0.0326) |
| West Midlands |  |  |  |  |  |  | 0.0172 |
| *(binary variable)* |  |  |  |  |  |  | (0.0273) |
| Yorkshire and the Humber |  |  |  |  |  |  | 0.0721*** |
| *(binary variable)* |  |  |  |  |  |  | (0.0266) |
| Constant | 0.191*** | 0.118*** | 0.116*** | 0.122*** | 0.117*** | 0.120*** | 0.0932*** |
|  | (0.00707) | (0.0187) | (0.0190) | (0.0208) | (0.0209) | (0.0210) | (0.0279) |
| Observations | 5,921 | 5,921 | 5,921 | 5,772 | 5,772 | 5,719 | 5,719 |
| R-squared | 0.256 | 0.259 | 0.259 | 0.256 | 0.257 | 0.261 | 0.263 |

*Note: Robust standard errors in parentheses; *** p<0.01, ** p<0.05, * p<0.1.*

## *Table S2.9. Results for different dependent variables regressed on Conservative vote*

| **Model** | **1** | **2** | **3** | **4** | **5** | **6** | **7** |
| --- | --- | --- | --- | --- | --- | --- | --- |
| **Dependent variable** | **Improved hygiene** | **Avoid crowded places** | **Wear face mask** | **Level of Covid fear** | **Avoid going to work** | **Quarantine tourists** | **Covid global view** |
| Voted Conservative at last General Election | -0.0597*** | -0.0573*** | -0.0742*** | 0.0437 | -0.0254** | -0.0831*** | -0.0918*** |
| *(binary variable)* | (0.0164) | (0.0160) | (0.0148) | (0.0411) | (0.00986) | (0.0143) | (0.0266) |
| Age respondent | 0.00185*** | 0.00639*** | 0.00496*** | -0.00405*** | -0.00290*** | 0.000832** | 0.00896*** |
|  | (0.000463) | (0.000449) | (0.000416) | (0.00114) | (0.000290) | (0.000414) | (0.000749) |
| Male respondent | -0.0790*** | -0.101*** | -0.0731*** | 0.0310 | 0.0222*** | -0.0659*** | 0.0421* |
| *(binary variable)* | (0.0135) | (0.0133) | (0.0122) | (0.0334) | (0.00854) | (0.0120) | (0.0222) |
| Number of Children | -0.0285*** | -0.0316*** | -0.0421*** | 0.0845*** | 0.00402 | 0.00347 | -0.0138 |
|  | (0.00996) | (0.00971) | (0.00963) | (0.0258) | (0.00729) | (0.00867) | (0.0165) |
| C2DE class | -0.00170 | -0.00712 | -0.0526*** | -0.00227 | -0.0775*** | 0.0603*** | -0.0370 |
| *(binary variable)* | (0.0143) | (0.0141) | (0.0130) | (0.0359) | (0.00771) | (0.0129) | (0.0237) |
| Voted Leave | -0.0674*** | -0.0517*** | -0.122*** | 0.102** | -0.0207** | 0.0521*** | -0.0556** |
| *(binary variable)* | (0.0166) | (0.0163) | (0.0151) | (0.0415) | (0.00962) | (0.0145) | (0.0273) |
| *Regional dummy variables (Ref: East Midlands)* |  |  |  |  |  |  |  |
| East of England | 0.0159 | 0.0174 | 0.0232 | 0.0121 | -0.00814 | 0.00593 | 0.00523 |
| *(binary variable)* | (0.0307) | (0.0305) | (0.0282) | (0.0763) | (0.0190) | (0.0280) | (0.0509) |
| London | 0.0164 | -0.00239 | 0.0760*** | 0.0656 | 0.0189 | -0.0453* | 0.0313 |
| *(binary variable)* | (0.0310) | (0.0303) | (0.0280) | (0.0784) | (0.0206) | (0.0273) | (0.0502) |
| North East | 0.0350 | 0.0105 | 0.0322 | 0.0482 | 0.00563 | 0.00247 | 0.0506 |
| *(binary variable)* | (0.0387) | (0.0375) | (0.0347) | (0.0967) | (0.0237) | (0.0345) | (0.0612) |
| North West | 0.00191 | 0.0526* | -0.00437 | 0.124 | 0.000618 | -0.00303 | -0.0216 |
| *(binary variable)* | (0.0306) | (0.0302) | (0.0284) | (0.0781) | (0.0193) | (0.0276) | (0.0501) |
| Scotland | -0.000666 | 0.0257 | 0.135*** | 0.105 | 0.0211 | -0.0180 | -0.0413 |
| *(binary variable)* | (0.0370) | (0.0362) | (0.0307) | (0.0909) | (0.0245) | (0.0335) | (0.0619) |
| South East | 0.00878 | 0.0420 | 0.0668** | 0.0246 | 0.0183 | -0.0465* | 0.00387 |
| *(binary variable)* | (0.0285) | (0.0281) | (0.0261) | (0.0707) | (0.0181) | (0.0254) | (0.0472) |
| South West | 0.0223 | 0.0589** | 0.0749*** | -0.0624 | 0.000305 | 0.0155 | 0.0369 |
| *(binary variable)* | (0.0302) | (0.0298) | (0.0273) | (0.0756) | (0.0188) | (0.0277) | (0.0506) |
| Wales | 0.0314 | 0.0156 | 0.120*** | 0.121 | -0.00448 | 0.0109 | 0.00466 |
| *(binary variable)* | (0.0392) | (0.0386) | (0.0346) | (0.100) | (0.0243) | (0.0359) | (0.0661) |
| West Midlands | -0.00894 | 0.00598 | -0.0135 | 0.116 | -0.0235 | -0.00232 | -0.0437 |
| *(binary variable)* | (0.0321) | (0.0312) | (0.0300) | (0.0826) | (0.0195) | (0.0287) | (0.0532) |
| Yorkshire and the Humber | -0.0251 | 0.00876 | -0.0288 | 0.105 | -0.0227 | -0.0377 | -0.0239 |
| *(binary variable)* | (0.0318) | (0.0314) | (0.0296) | (0.0811) | (0.0194) | (0.0284) | (0.0524) |
| Constant | 1.480*** | 1.242*** | 1.544*** | 3.417*** | 1.309*** | 1.309*** | 2.667*** |
|  | (0.0341) | (0.0335) | (0.0316) | (0.0853) | (0.0229) | (0.0306) | (0.0556) |
| Observations | 5,489 | 5,489 | 5,489 | 5,967 | 5,489 | 5,967 | 5,967 |
| R-squared | 0.022 | 0.056 | 0.075 | 0.009 | 0.057 | 0.019 | 0.029 |

*Note: Robust standard errors in parentheses; *** p<0.01, ** p<0.05, * p<0.1.*

## *Table S2.10. Results when adding more controls to regression on vaccination status (at least one dose)*

| **Model** | **1** | **2** | **3** | **4** | **5** | **6** | **7** |
| --- | --- | --- | --- | --- | --- | --- | --- |
| Voted Conservative at last General Election | 0.0153** | 0.0149** | 0.0188*** | 0.0126* | 0.0137** | 0.0139** | 0.0159** |
| *(binary variable)* | (0.00667) | (0.00668) | (0.00665) | (0.00655) | (0.00671) | (0.00656) | (0.00652) |
| Age respondent | 0.00163*** | 0.00148*** | 0.00131*** | 0.00173*** | 0.00178*** | 0.00173*** | 0.00142*** |
|  | (0.000215) | (0.000215) | (0.000208) | (0.000210) | (0.000220) | (0.000210) | (0.000206) |
| Male respondent | 0.000494 | 0.000909 | 0.00322 | -0.00292 | -0.00316 | -0.00194 | -0.00450 |
| *(binary variable)* | (0.00551) | (0.00559) | (0.00552) | (0.00546) | (0.00554) | (0.00548) | (0.00542) |
| Number of Children | -0.00980* | -0.00981* | -0.00758 | -0.0112** | -0.0110* | -0.0114** | -0.0109** |
|  | (0.00558) | (0.00563) | (0.00551) | (0.00529) | (0.00562) | (0.00526) | (0.00515) |
| C2DE class | -0.0346*** | -0.0344*** | -0.0305*** | -0.0327*** | -0.0323*** | -0.0337*** | -0.0314*** |
| *(binary variable)* | (0.00663) | (0.00663) | (0.00650) | (0.00658) | (0.00668) | (0.00659) | (0.00649) |
| Voted Leave | -0.0405*** | -0.0413*** | -0.0335*** | -0.0471*** | -0.0424*** | -0.0481*** | -0.0453*** |
| *(binary variable)* | (0.00707) | (0.00709) | (0.00687) | (0.00698) | (0.00713) | (0.00703) | (0.00685) |
| Improved hygiene | 0.0380*** |  |  |  |  |  |  |
|  | (0.00526) |  |  |  |  |  |  |
| Avoid crowded places |  | 0.0339*** |  |  |  |  |  |
|  |  | (0.00553) |  |  |  |  |  |
| Wear face mask |  |  | 0.0782*** |  |  |  |  |
|  |  |  | (0.00764) |  |  |  |  |
| Level of Covid-19 fear |  |  |  | -0.00220 |  |  |  |
|  |  |  |  | (0.00215) |  |  |  |
| Avoid going to work |  |  |  |  | 0.0293*** |  |  |
|  |  |  |  |  | (0.00726) |  |  |
| Quarantine tourists |  |  |  |  |  | 0.0159*** |  |
|  |  |  |  |  |  | (0.00564) |  |
| Covid-19 global view |  |  |  |  |  |  | 0.0360*** |
|  |  |  |  |  |  |  | (0.00400) |
| *Regional dummy variables (Ref: East Midlands)* | |  |  |  |  |  |  |
| East of England | 0.00286 | 0.00288 | 0.00165 | 0.00108 | 0.00370 | 0.000963 | 0.000869 |
| *(binary variable)* | (0.0131) | (0.0131) | (0.0129) | (0.0126) | (0.0131) | (0.0126) | (0.0125) |
| London | 0.00292 | 0.00362 | -0.00240 | -0.00138 | 0.00299 | -0.000804 | -0.00265 |
| *(binary variable)* | (0.0131) | (0.0131) | (0.0129) | (0.0127) | (0.0131) | (0.0127) | (0.0126) |
| North East | 0.0216 | 0.0226 | 0.0205 | 0.0174 | 0.0228 | 0.0173 | 0.0155 |
| *(binary variable)* | (0.0139) | (0.0138) | (0.0137) | (0.0138) | (0.0139) | (0.0139) | (0.0138) |
| North West | 0.00573 | 0.00401 | 0.00614 | 0.00188 | 0.00578 | 0.00166 | 0.00239 |
| *(binary variable)* | (0.0128) | (0.0128) | (0.0126) | (0.0123) | (0.0128) | (0.0123) | (0.0122) |
| Scotland | -0.00232 | -0.00321 | -0.0129 | -0.0110 | -0.00296 | -0.0109 | -0.00973 |
| *(binary variable)* | (0.0154) | (0.0155) | (0.0152) | (0.0155) | (0.0155) | (0.0155) | (0.0155) |
| South East | -0.00555 | -0.00664 | -0.0104 | -0.00837 | -0.00575 | -0.00769 | -0.00857 |
| *(binary variable)* | (0.0123) | (0.0123) | (0.0121) | (0.0118) | (0.0123) | (0.0118) | (0.0117) |
| South West | 0.00456 | 0.00341 | -0.000456 | 0.000507 | 0.00540 | 0.000397 | -0.000685 |
| *(binary variable)* | (0.0126) | (0.0126) | (0.0124) | (0.0122) | (0.0126) | (0.0122) | (0.0121) |
| Wales | 0.00432 | 0.00498 | -0.00388 | -0.0120 | 0.00564 | -0.0124 | -0.0124 |
| *(binary variable)* | (0.0168) | (0.0168) | (0.0165) | (0.0176) | (0.0168) | (0.0176) | (0.0173) |
| West Midlands | 0.00565 | 0.00511 | 0.00637 | -0.00395 | 0.00600 | -0.00417 | -0.00263 |
| *(binary variable)* | (0.0135) | (0.0135) | (0.0134) | (0.0133) | (0.0135) | (0.0133) | (0.0131) |
| Yorkshire and the Humber | 0.00539 | 0.00414 | 0.00668 | 0.00192 | 0.00510 | 0.00229 | 0.00255 |
| *(binary variable)* | (0.0136) | (0.0136) | (0.0134) | (0.0130) | (0.0137) | (0.0130) | (0.0129) |

*Note: Robust standard errors in parentheses; *** p<0.01, ** p<0.05, * p<0.1.*

## *Table S2.11. Results for different operationalization of vaccination status*

| **Model** | **1** | **2** | **3** | **4** |
| --- | --- | --- | --- | --- |
| **Dependent variable** | **Will not get vaccinated (coded 1, 0 otherwise)** | **Vaccine hesitant (coded 1, 0 otherwise)** | **Vaccinated or planning to get vaccinated (coded 1, 0 otherwise)** | **Not vaccinated and no plan to get vaccinated (coded 1, 0 otherwise)** |
| Conservative voter | -0.00975* | -0.00137 | 0.0111* | -0.0111* |
|  | (0.00529) | (0.00328) | (0.00611) | (0.00611) |
| Age respondent | -0.000588*** | -0.000553*** | 0.00114*** | -0.00114*** |
|  | (0.000157) | (0.000115) | (0.000191) | (0.000191) |
| Male respondent | 0.00485 | 0.000165 | -0.00502 | 0.00502 |
| *(binary variable)* | (0.00425) | (0.00290) | (0.00505) | (0.00505) |
| Number of Children | 0.00517 | 0.000217 | -0.00539 | 0.00539 |
|  | (0.00401) | (0.00263) | (0.00465) | (0.00465) |
| C2DE class | 0.0129*** | 0.0100*** | -0.0229*** | 0.0229*** |
| *(binary variable)* | (0.00483) | (0.00365) | (0.00592) | (0.00592) |
| Voted Leave | 0.0393*** | 0.00799** | -0.0473*** | 0.0473*** |
| *(binary variable)* | (0.00559) | (0.00342) | (0.00642) | (0.00642) |
| Covid global view | -0.0263*** | -0.00767*** | 0.0340*** | -0.0340*** |
|  | (0.00350) | (0.00178) | (0.00380) | (0.00380) |
| *Regional dummy variables (Ref: East Midlands)* |  |  |  |  |
| East of England | 0.00118 | -0.000110 | -0.00107 | 0.00107 |
| *(binary variable)* | (0.0102) | (0.00641) | (0.0119) | (0.0119) |
| London | -0.00708 | 0.00527 | 0.00182 | -0.00182 |
| *(binary variable)* | (0.00942) | (0.00715) | (0.0115) | (0.0115) |
| North East | -0.0167 | 0.00305 | 0.0136 | -0.0136 |
| *(binary variable)* | (0.0102) | (0.00839) | (0.0130) | (0.0130) |
| North West | 0.00276 | -0.00683 | 0.00407 | -0.00407 |
| *(binary variable)* | (0.0101) | (0.00547) | (0.0113) | (0.0113) |
| Scotland | -0.00131 | -0.000245 | 0.00156 | -0.00156 |
| *(binary variable)* | (0.0116) | (0.00743) | (0.0135) | (0.0135) |
| South East | 0.00305 | 0.00359 | -0.00665 | 0.00665 |
| *(binary variable)* | (0.00945) | (0.00619) | (0.0110) | (0.0110) |
| South West | -0.00661 | 0.00317 | 0.00345 | -0.00345 |
| *(binary variable)* | (0.00932) | (0.00655) | (0.0112) | (0.0112) |
| Wales | 0.0147 | -0.00395 | -0.0107 | 0.0107 |
| *(binary variable)* | (0.0148) | (0.00729) | (0.0163) | (0.0163) |
| West Midlands | -0.00423 | 0.00850 | -0.00428 | 0.00428 |
| *(binary variable)* | (0.0102) | (0.00770) | (0.0125) | (0.0125) |
| Yorkshire and the Humber | -0.00545 | 0.000425 | 0.00503 | -0.00503 |
| *(binary variable)* | (0.0101) | (0.00674) | (0.0119) | (0.0119) |
| Constant | 0.120*** | 0.0577*** | 0.822*** | 0.178*** |
|  | (0.0158) | (0.0101) | (0.0182) | (0.0182) |
| Observations | 5,967 | 5,967 | 5,967 | 5,967 |
| R-squared | 0.038 | 0.014 | 0.048 | 0.048 |

*Note: Robust standard errors in parentheses; *** p<0.01, ** p<0.05, * p<0.1.*
